# Scaling-Up Microwave-Assisted Synthesis of Highly Defective Pd@UiO-66-NH_2_ Catalysts for Selective Olefin Hydrogenation under Ambient Conditions

**DOI:** 10.1021/acsami.4c03106

**Published:** 2024-04-26

**Authors:** Raúl M. Guerrero, Ignacio D. Lemir, Sergio Carrasco, Carlos Fernández-Ruiz, Safiyye Kavak, Patricia Pizarro, David P. Serrano, Sara Bals, Patricia Horcajada, Yolanda Pérez

**Affiliations:** † Advanced Porous Materials Unit, IMDEA Energy Institute, Avda. Ramón de la Sagra, 3, Móstoles 28935, Madrid, Spain; ‡ Thermochemical Processes Unit, IMDEA Energy Institute, Avda. Ramón de la Sagra, 3, Móstoles 28935, Madrid, Spain; § EMAT and NANOlab Center of Excellence, University of Antwerp, Groenenborgerlaan 171, Antwerp 2020, Belgium; ∥ Chemical and Environmental Engineering Group, Rey Juan Carlos University, C/Tulipán, s/n, Móstoles 28933, Madrid, Spain; ⊥ COMET-NANO Group, ESCET, Universidad Rey Juan Carlos, C/Tulipán, s/n, Móstoles 28933, Madrid, Spain

**Keywords:** microwave-assisted synthesis, defect engineering, UiO-66-NH_2_, gram-scale, selective hydrogenation, palladium

## Abstract

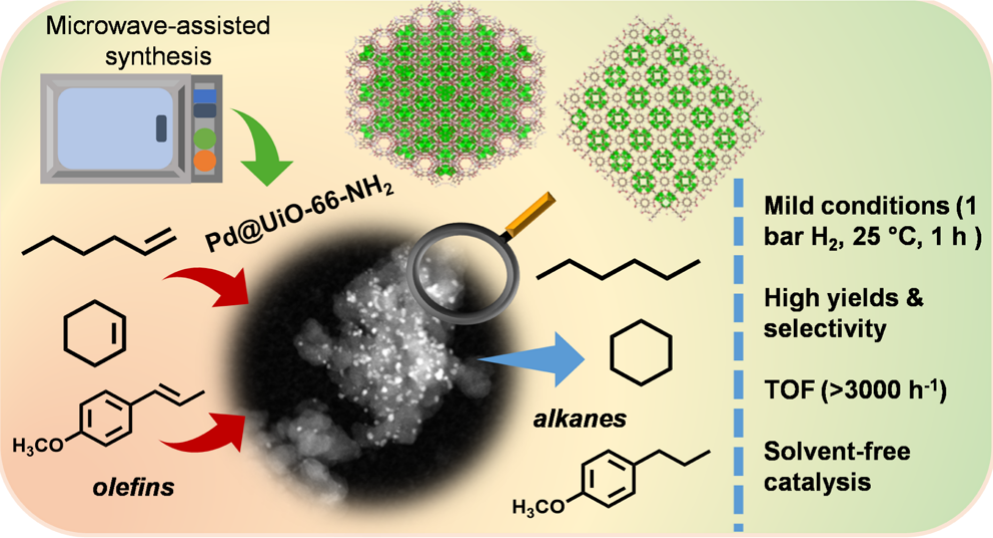

The need to develop green and cost-effective industrial catalytic processes has led to growing interest in preparing more robust, efficient, and selective heterogeneous catalysts at a large scale. In this regard, microwave-assisted synthesis is a fast method for fabricating heterogeneous catalysts (including metal oxides, zeolites, metal–organic frameworks, and supported metal nanoparticles) with enhanced catalytic properties, enabling synthesis scale-up. Herein, the synthesis of nanosized UiO-66-NH_2_ was optimized via a microwave-assisted hydrothermal method to obtain defective matrices essential for the stabilization of metal nanoparticles, promoting catalytically active sites for hydrogenation reactions (760 kg·m^–3^·day^–1^ space time yield, STY). Then, this protocol was scaled up in a multimodal microwave reactor, reaching 86% yield (ca. 1 g, 1450 kg·m^–3^·day^–1^ STY) in only 30 min. Afterward, Pd nanoparticles were formed *in situ* decorating the nanoMOF by an effective and fast microwave-assisted hydrothermal method, resulting in the formation of Pd@UiO-66-NH_2_ composites. Both the localization and oxidation states of Pd nanoparticles (NPs) in the MOF were achieved using high-angle annular dark-field scanning transmission electron microscopy (HAADF-STEM) and X-ray photoelectron spectroscopy (XPS), respectively. The optimal composite, loaded with 1.7 wt % Pd, exhibited an extraordinary catalytic activity (>95% yield, 100% selectivity) under mild conditions (1 bar H_2_, 25 °C, 1 h reaction time), not only in the selective hydrogenation of a variety of single alkenes (1-hexene, 1-octene, 1-tridecene, cyclohexene, and tetraphenyl ethylene) but also in the conversion of a complex mixture of alkenes (i.e., 1-hexene, 1-tridecene, and anethole). The results showed a powerful interaction and synergy between the active phase (Pd NPs) and the catalytic porous scaffold (UiO-66-NH_2_), which are essential for the selectivity and recyclability.

## 1 Introduction

At the moment, 90% of the chemicals require the presence of a catalyst during their industrial production,^1^ being of extreme importance in the case of pharmaceuticals or refined petrochemicals. Efforts are currently focused on the design of more efficient and economically profitable methodologies.^2^ However, the integration of such a catalyst in industrial processes is not exempt of relevant limitations, e.g., catalyst passivation and/or poisoning, aspects with a dramatic impact over their lifetime and recyclability, particularly when moving from batch to continuous operation.

Catalytic hydrogenation is one of the most widespread reactions within the industry because of the extensive range of chemical products that can be obtained, including fine chemicals, fuels, or fibers, among others.^3^ In particular, the reduction of olefins has been broadly studied upon adding metal-based homogeneous catalysts, including Ni,^4^ Pd,^5^ and Pt,^6^ in the presence of hydrogen donors. However, several aspects related to their recyclability, cost, and/or toxicity have hampered their use in industrial processes. Nowadays, large-scale hydrogenation is typically performed in the presence of palladium-based composites such as Pd/C and Pd/Al_2_O_3_^7^ since Pd nanoparticles (Pd NPs) exhibit an exceptional catalytic activity and high chemical reactivity.^8^ In such materials, the low stability and limited recyclability of isolated Pd NPs,^9^ ascribed to their aggregation, have been partially circumvented by immobilizing them within convenient porous supports.^10^ In this sense, different Pd-based composites have been studied in the last years in order to improve the overall catalytic performance, minimizing the metal content, preventing metal leaching, and finding the optimal synergy between the catalyst itself and the support. The ultimate challenge has revealed to be the passivation of the Pd surface and the progressive obstruction of diffusion channels in porous materials hampering the accessibility of chemicals toward metal binding sites. Besides, the principles of the green chemistry should be observed, i.e., novel synthetic procedures must be scalable at an industrial level. Thus, there is an urgent need for (i) the preparation of convenient heterogeneous catalysts in the form of composites to reduce the catalyst loading and improve recyclability and (ii) a large-scale synthetic protocol minimizing the fabrication cost by decreasing reaction times and making a better use of the starting materials.

In this context, metal–organic frameworks (MOFs), comprising organic linkers and metal ions, have emerged as potential catalytic supports thanks to their inherent porosity and the presence of coordinatively unsaturated sites (CUSs), allowing for a fast diffusion of chemicals and their interaction with active sites, thus decreasing the energy barrier of a broad variety of reactions.^11^ These hybrid polymers have been used in different manners for catalysis: (a) as-synthesized, because of the presence of CUS within the structure, typically acting as Lewis/Brønsted acidic sites;^12^ (b) introducing new open metal sites and transition metal complexes by pre- or postsynthetic modification of organic linkers or MOFs (i.e., organic and inorganic parts), respectively;^13^ and (c) by loading catalytically active metal cations inside the porous cages and, if necessary, followed by their reduction to metal colloids that remain confined within the pores.^14^ Several authors have reported a synergistic catalytic performance of MOF-based composites when metal nanoparticles are included, due to any of the following effects: (a) the activity of the structural metal itself is enhanced, (b) the accessibility of chemicals to the new metal sites is improved, or (c) a singular spatial rearrangement of the chemicals anchored within the pores is displayed that cannot be observed otherwise. Moreover, nucleation and growth of such metal nanoparticles are extraordinarily controlled because of the confinement effect attributed to the pores, which, in addition, provide further stability to the colloid.^15^

The role of defects in MOFs has been demonstrated to be of extreme relevance for boosting the catalytic performance.^16^ In particular, hydrogenation reactions have shown remarkable improvements when using defective matrices.^17^ Despite being proposed in the late 2000s, defect engineering irrupted in MOF technology by the mid-2010s, when hundreds of unexplored structures appeared, allowing for significant structural modifications without remarkable collapse.^18^ Since then, several strategies to generate defects have been proposed, including modifications in the synthesis (mechanochemical, sonochemical), pre- and postsynthetic modifications, chemical etching, or the use of surfactants, among others.^18−20^

From the extensive plethora of available MOFs, the microporous zirconium-terephthalate UiO-66 (*Universitetet i Oslo*) has attracted a great deal of attention from the catalytic community^21,22^ due to its facile synthesis, high stability, and relevant physicochemical properties, whose origin relies on the Zr–O-based node despite the high coordination of the cluster.^23^ Interestingly, catalysis can be promoted not only through Brønsted acid sites in the nodes^24^ but also upon *in situ* reduction of cations, yielding metal colloids within the pores. Inducing defects in the UiO-66 matrix has been also extensively studied, particularly by using different acidic modulators and microwave radiation.^25−27^ Typically, monodentate carboxylates occupy linker positions during the MOF synthesis, thus resulting in missing linker positions in the material. UiO-66 shows a wide flexibility in terms of defect generation, even allowing up to 67% ligand replacement without collapsing.^28^ These defects considerably attract Pd(II) cations that can easily diffuse throughout the network within a few seconds by a simple wet impregnation.^29^ On the other hand, the use of 2-aminoterephthalic acid as the organic ligand (i.e. UiO-66-NH_2_) enhances, even more, the stability of Pd species with the aid of –NH_2_ moieties.^30^ Besides, the use of microwave radiation brings into scene the additional benefit of improving phase purity and yields by decreasing reaction time and particle size, aside from introducing a larger amount of defects,^31,32^ all of them critical aspects in catalysis.

However, there are only a few examples in the literature reporting on the use of metal NPs@UiO-66-NH_2_ for the hydrogenation of alkenes and aromatic hydrocarbons.^33^ In 2014, Huang et al.^34^ solvothermally prepared the Pt@UiO-66-NH_2_ composite in 24 h, which showed a limited catalytic activity in the hydrogenation of light olefins at 35 °C and 1 bar H_2_ (66% conversion of cyclooctene after 24 h). Ning et al.^35^ reported the solvothermal synthesis of Pd@UiO-66 and its further Pd impregnation, obtaining the composite after 48 h, which was studied for phenol hydrogenation (100% conversion, at 20 bar H_2_ and 60 °C for 16 h), lacking specific mention to the catalyst stability. A similar procedure was reported by Li et al.^36^ for the preparation of Pd@UiO-66-NH_2_, which also displayed a high styrene conversion (91%) but at 50 °C and for 15 h. More recently, Liu et al.^37^ prepared a Pt/UiO-66-NH_2_ composite via solvothermal synthesis in the presence of poly(vinylpyrrolidone) (PVP)-stabilized Pt NPs, achieving a low activity in the hydrogenation of cyclooctene and *trans*-stilbene (ca. 50% conversion) at 35 °C and 1 atm H_2_ after 24 h. Despite these interesting results, reported methods are not time- and cost-efficient.

In the present work, we have prepared a Pd@UiO66-NH_2_ composite using a more effective microwave-assisted hydrothermal route (1.5 h vs. 24–48 h in reported conventional solvothermal methodologies), even achieving full conversion with less severe reaction conditions (1 bar H_2_, room temperature, 1 h reaction time). Moreover, we have (i) optimized the synthesis protocol of UiO-66-NH_2_ using a monomodal microwave instrument (91 ± 5% yield in only 20 min reaction time) to find the optimal candidate for catalytic purposes in terms of textural properties, particle size, stability, and defects; (ii) scaled up this procedure in a multimodal microwave reactor (gram-scale, 84 ± 3% yield), and after a wet impregnation with Pd(II) and a fast microwave-assisted reduction step (150 °C for 10 min), the Pd@UiO-66-NH_2_ composite was obtained and characterized (Pd NP size of 3–4 nm); (iii) successfully used the MOF composite as a catalyst for the selective hydrogenation of olefins (including hydrogenation of a mixture of three alkenes), under mild conditions (1 atm H_2_, 25 °C, 1 h); and (iv) studied the interaction and the synergistic effect between the Pd NPs and the MOF scaffold by using high-angle annular dark-field scanning transmission electron microscopy (HAADF-STEM) and X-ray photoelectron spectroscopy (XPS).

## 2 Experimental Section

### 2.1 Synthesis

#### 2.1.1 Materials

All chemicals were purchased from commercial companies and used without purification: zirconium(IV) oxychloride octahydrate (ZrOCl_2_·8H_2_O, Sigma-Aldrich, 99.5%), zirconium chloride (ZrCl_4_, Sigma-Aldrich, 99.5%) and zirconium propoxide (Zr(OPr)_4_, Sigma-Aldrich 70 wt % in 1-propanol), palladium(II) chloride (PdCl_2_, Merck, 99%), sodium borohydride (NaBH_4_, Sigma-Aldrich, 98%), 2-aminoterephthalic acid (2ATA, Acros Organics, 99%), *N*,*N*-dimethylformamide (DMF, Chemlab, 99%), trifluoracetic acid (TFA, ACROS Organics, 99%), acetic acid glacial (Supelco), formic acid (Thermo Scientific, 98%), absolute ethanol (Molecular Biology grade, Fisher), acetone (HPLC grade, Chem-lab), and poly(vinylpyrrolidone) (PVP, MW = 30,000, AR).

For comparative purposes, PVP-stabilized Pd nanoparticles (PVP@Pd NPs) were prepared according to a microwave-assisted method reported elsewhere^38^ and Pd/Al_2_O_3_ was purchased from Sigma-Aldrich.

#### 2.1.2 Synthesis of UiO-66-NH_2_

Fabrication of nanometric UiO-66-NH_2_ crystals was performed in a monomodal microwave instrument (Anton Paar Monowave 300) by adapting a protocol described in the literature,^39^ with some minor modifications. Optimization tests can be found in Table S1. Selection of the optimal MOF was performed after the analysis of the candidates by powder X-ray diffraction (PXRD), thermogravimetric analysis (TGA), and nitrogen sorption. The optimal synthetic protocol for the monomodal instrument is described as follows: ZrOCl_2_·8H_2_O (296 mg; 0.92 mmol) and 2ATA (167 mg; 0.92 mmol) were dissolved in 10 mL of DMF inside a 30 mL glass microwave vial. TFA (0.71 mL; 9.23 mmol) was added to the mixture, and then, the vial was placed in the microwave instrument. Synthesis consisted of three steps: (i) a ramp to reach 175 °C, from room temperature, in 5 min; (ii) keeping 175 °C for 20 min (max. pressure: 14.0 bar, 15–20 W to maintain the temperature in the form of pulsed irradiation); and (iii) cooling to 60 °C with an air flow. The pale-yellow suspension was centrifuged (12,000 rpm, 15 min), and the gel-like solid was washed by resuspension using fresh DMF (30 mL, ×3) and absolute ethanol (30 mL, ×3), under similar centrifugation conditions. The solid was transferred to a 25 mL glass vial and dried for 12 h at 100 °C to obtain a yellowish material with the consistency of a foam (316.7 mg; 91 ± 5% yield (*n* = 5)) on metal basis considering the structural formula Zr_6_O_4_(OH)_4_(2ATA)_5_(TFA)_2_(DMF)_4_ with MW = 2091.4 g·mol^–1^, in agreement with the TGA results. The space time yield (STY) was estimated to be 760 kg·m^–3^·day^–1^.

The material was scaled up (×3.3) afterward in a multimodal microwave instrument (One Touch Technology Mars6 240/50). Briefly, ZrOCl_2_·8H_2_O (980 mg; 3.04 mmol) and 2-ATA (540 mg; 2.98 mmol) were dissolved in 33 mL of DMF inside a 45 mL microwave Teflon-lined reactor. TFA (2.34 mL; 30.58 mmol) was then added to the mixture, and the reaction proceeded in the same manner as described above. A similar procedure was followed in order to wash and activate the material. The yield (metal basis) was 84 ± 3% (*n* = 5) (908.6 mg), lower than that obtained with the monomodal reactor but with a higher STY (1450 kg·m^–3^·day^–1^).

#### 2.1.3 Synthesis of Pd@UiO-66-NH_2_

The preparation of Pd@UiO-66-NH_2_ was performed by wet impregnation of the metal precursor, followed by a chemical reduction in one pot using the previous multimodal microwave instrument. UiO-66-NH_2_ (83.00 mg, 0.04 mmol), PdCl_2_ (2 or 5 wt % Pd), and Mili-Q water (10 mL) were added to a Teflon-lined reactor. The mixture was kept under magnetic stirring for 30 min, and then, 1 mL of a solution consisting of NaBH_4_ (3 mg·mL^–1^ in MeOH) was added dropwise under vigorous stirring for 20 min. Finally, the resulting suspension was heated using a 5 min ramp from 25 to 150 °C and maintained for 10 min. After cooling, the composite was recovered as a grayish solid by filtration (nylon, 0.22 μm) and washed several times with deionized water. The resulting materials were denoted as Pd(1.7%)@UiO-66-NH_2_ and Pd(4.7%)@UiO-66-NH_2_ containing 1.7 and 4.7 wt % of Pd, respectively.

### 2.2 Hydrogenation Reactions

The activity and selectivity of the as-prepared composites were studied in hydrogenation reactions of different alkenes (including 1-hexene, 1-octene, 1-tridecene, cyclohexene, and tetraphenyl ethylene), using a batch-pressurized stainless-steel poly(tetrafluoroethylene) (PTFE)-lined reactor (100 mL, miniclave from Buchiglas), at 1 bar (gas, H_2_, 99.9998%) and 25 °C. 10 mg of catalyst was dispersed in alkene solution (5 mmol in 25 mL of acetyl acetate). The reactor was purged three times prior to the reaction by increasing the H_2_ pressure to 1 bar and opening the output valve. Then, the reactor was pressurized once again with 1 bar H_2_ and the reaction was considered to begin when magnetic stirring started. After 1 h, the stirring was stopped and the output valve was opened, ending the reaction. The composite was recovered by filtration and dried at 80 °C in a furnace for 3 h. The resulting solution was analyzed by gas chromatography–mass spectrometry (GC–MS) (Agilent 8860 GC-5977B GC/MSD equipped with a HP5-MS UI column 30 m × 0.25 mm × 0.25 mm), and the concentration of all products and byproducts obtained was calculated with an external calibration, using cyclohexanol as an internal standard.

### 2.3 Characterization

Powder X-ray diffraction (PXRD) patterns were acquired in a PANalytical Empyrean powder diffractometer (PANalytical, Lelyweg, The Netherlands) with Cu Kα = 1.5406 Å in reflection mode with a 2θ scan between 3 and 50° (MOFs) and 3 and 90° (composite) with a step of 0.013° and a scanning speed of 0.1°·s^–1^. Fourier-transform infrared spectra (FTIR) were collected in a Nicolet 6700 (Thermo Scientific) in attenuated total reflectance (ATR) mode using a diamond accessory. Spectra were obtained with 32 scans and a resolution of 4 cm^–1^, within 4000–400 cm^–1^. Thermogravimetric analysis (TGA) was carried out in an SDT Q-600 thermobalance (TA Instruments, New Castle, DE) with a heating profile from room temperature to 800 °C, under oxidative conditions (air; 100 mL·min^–1^) and a heating rate of 5 °C·min^–1^. Inductively coupled plasma optical emission spectroscopy (ICP-OES) was performed in a PerkinElmer Optima 7300 DV (samples were previously digested using a piranha solution). Nitrogen sorption isotherms were acquired in a Micromeritics Tristar II PLUS at 77 K after activating the samples (ca. 50–100 mg) at 150 °C for 16 h under primary vacuum. The specific surface area was calculated according to the Brunauer–Emmett–Teller (BET) equation in the relative pressure range *P*/*P*_0_ = 0.01–0.30. Pore size distribution was estimated with the Horvath–Kawazoe (HK) method (using a spherical model), and pore volume and pore area were estimated with the *t*-plot method in the relative pressure range *P*/*P*_0_ = 0.01–0.80. Field emission scanning electron microscopy (FE-SEM) images were obtained in a JEOL (JSM 7900F) scanning microscope at an accelerating voltage of 10 kV. Samples for transmission electron microscopy (TEM) were prepared by casting a drop of solution onto a Cu TEM grid coated with amorphous carbon. Routine TEM images were acquired using a JEOL microscope (JEM 1400 FLASH) with 200 kV acceleration voltage with a secondary detector to perform energy dispersive X-ray spectroscopy (EDS) measurements for elemental analysis. To acquire high-angle annular dark-field scanning TEM (HAADF–STEM) images, an aberration-corrected Thermo Fisher Scientific Titan Cubed electron microscope, operated at 300 kV, was used. The camera length was set at 230 mm, and the probe convergence semiangle was 15 mrad, yielding inner and outer HAADF detector collection semiangles of 26 and 155 mrad, respectively.

## 3 Results and Discussion

Defects in MOFs are essential for catalysis and can be introduced throughout/within the matrices in different manners, including (i) nanosizing (higher exposure of metal active sites); (ii) generation of defective positions around CUSs by using modulators; and (iii) formation of novel CUSs by ligand modification or cation grafting, which can be additionally reduced to originate colloidal aggregates.^16,17,40,41^ In this sense, UiO-66-NH_2_ was chosen because it^42^ (a) shows extraordinary thermal and chemical robustness under harsh conditions (reported thermal decomposition temperature values from 425 to 500 °C); (b) allows an extensive inclusion of defects and chemical/physical modifications; and (c) carries amino moieties capable of interacting with other metal species, acting as secondary metal active sites. Several authors have demonstrated the superior catalytic performance of UiO-66 when downsizing the particle size below 100 nm, acquiring a gel-like consistency for particles <30 nm.^43^ This aspect is of extreme relevance since such gels can be easily shaped through different methodologies, being promising candidates as catalysts because of the high tolerance of this framework when generating defects (up to 67% ligands can be removed) without suffering structural damage, which also circumvents mass transfer limitations. Note that despite UiO-66-NH_2_ is a robust material in terms of fabrication and stability in a broad range of harsh conditions, the compactness of the framework and the high coordination of the Zr-cluster may prevent the accessibility and coordination of reagents to catalytically active sites.^44,45^ Both “missing linker” and “missing metal cluster” defects (with respect to the ideal UiO-66 structure) exist as a result of terephthalate ligands being replaced by other coordinative molecules.^46^ Indeed, the number of defects found in UiO-66 crystals and its analogues depends on the synthetic conditions, where modulators play a leading role.^18,39,47^

### 3.1 Microwave-Assisted Synthesis of UiO-66-NH_2_

Considering the above-mentioned premises, we evaluated three reaction parameters in the synthesis of UiO-66-NH_2_: nature and amount of the modulator and reaction time. The optimal candidate should be the one providing a compromise between crystallinity (homogeneity of physicochemical properties distributed alongside the whole extent of the material), particle size (easy manipulation and separation; high CUS exposure), stability and surface area (high adsorption capacity for metal cations while facilitating reagents’ diffusion), and production of the largest amount of MOFs in the shortest reaction time. Thus, 12 different syntheses were performed in a monomodal microwave instrument (Table S1), screening the resulting UiO-66-NH_2_ materials by means of their crystal size (dynamic light scattering (DLS) and PXRD, Figure S1), decomposition temperature and number of defects (TGA, Figure S2), BET surface area (Figure S3), and IR spectroscopy (Figure S4) as a function of both the modulator (amount of TFA; 0, 0.32, 0.62, 0.86, and 1.19 M) and reaction time (5 or 20 min) (Table S1).

Regarding the acidity of the modulator, we selected TFA for the synthesis. Although previously discarded by other authors because of the crystal aggregation,^47^ TFA was here the acid providing the largest amount of network defects and BET surface area under the fixed reaction conditions tested (175 °C, 1:1 metal/ligand mol/mol in 10 mL of DMF), maintaining the structural stability in agreement with previous observations.^48,49^ Stronger modulators, i.e., those acids showing low p*K*_a_ values, remain attached to the metal cluster during the polymer synthesis, providing additional “missing linker” positions after material activation. Weaker acids such as acetic acid (p*K*_a_ = 4.76; Table S1, entry 5) or formic acid (p*K*_a_ = 3.77), with a similar p*K*_a_ to the linker (3.51, 4.82), have resulted in larger but less defective crystals because of the capacity of the ligand to replace them.^44^ Other Zr(IV) precursors were also tested with similar (in the case of Zr(OPr)_4_) or poorer results (when using ZrCl_4_) (Table S1, entries 4, 11 and 12). However, in the case of the alkoxide, the matrix acquired a higher compactness and periodicity after 20 min, as revealed from the defect calculation (Table S1), pointing to a linker excess. Something similar occurred when using the chloride precursor, showing a lower number of linker defects than that obtained with TFA and, in this case, significantly decreasing the BET surface area (from 937 to 182 m^2^·g^–1^), suggesting that the linkers remain attached to the framework either onto the MOF surface or within the porosity but not occupying their natural crystallographic position.

**Figure 1:**
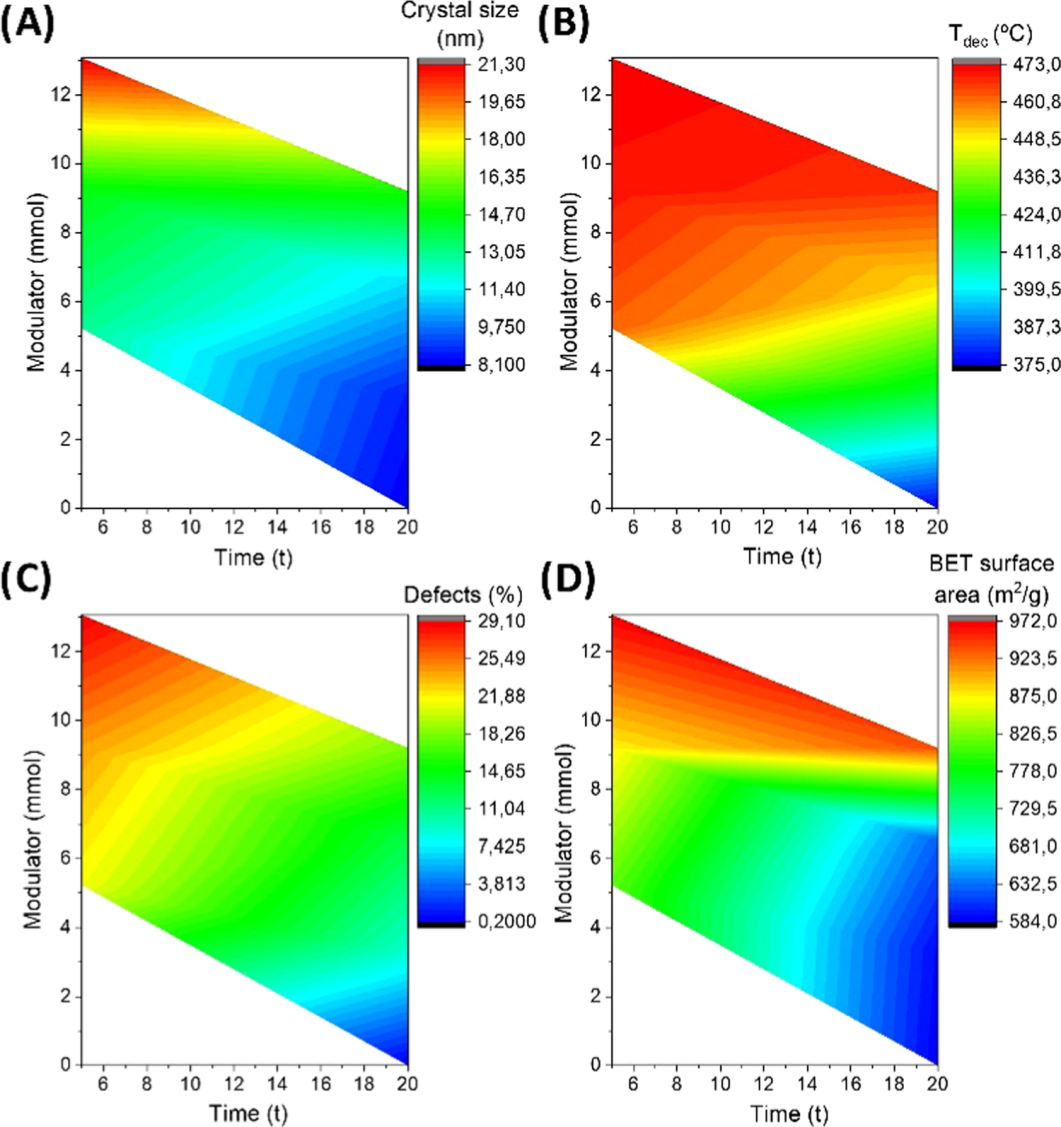
Density contour plots of (a) crystal size (estimated by using the Scherrer equation from PXRD); (b) decomposition temperature (first derivative of TGA curves); (c) number of defects (TGA, compared to theoretical weights at different temperatures; in terms of missing linker units); and (d) BET surface area (using nitrogen sorption isotherms), as a function of modulator concentration and reaction time. Red indicates the highest values, while blue indicates the opposite.

To better observe the influence of the different parameters, contour density maps of crystal size, decomposition temperature, number of defects, and BET surface area were represented as a function of both the modulator and the reaction time (Figure 1). Horizontally extended color regions denote that reaction time had little influence over the studied parameters, except when using a low concentration of TFA. In the absence of modulators, crystalline domain size (calculated from the (111) diffraction peak by using the Scherrer equation)^47^ decreased, probably because of the chemical etching of nuclei being indiscriminately attacked by basic species from DMF decomposition (dimethylamine) under microwave radiation.^50,51^ As a consequence, those crystals showed the lowest thermal stability, number of defects, and BET surface area. Note here that the defects evaluated in this work are related to the internal structure of the UiO-66-NH_2_ network in terms of missing linkers, thus being estimated from TGA residues.^27,28,52^ The addition of acidic modulators partially mitigated this phenomenon, resulting in a progressive color plateau as a function of time (*x*-axis) after increasing modulator concentration (*y*-axis). In fact, modulators accomplished here two distinctive roles, as defect inducers and base scavengers, preventing the attack from basic species. Color changes were more evident along the *y*-axis, i.e., TFA concentration modified the final features of such MOFs with a higher impact than time, particularly when the amount of the modulator used in their synthesis was enough (ratio >5.7:1 modulator:linker, mol/mol).

The highest defect concentration was found in the MOF showing both the best crystallinity (narrower diffraction peaks) and a large crystalline domain size. Decomposition temperatures suggested that the larger the number of defects, the higher the resistance toward heating. Other authors have reported the opposite behavior in UiO-66-NH_2_ networks, as one should have expected.^27^ We hypothesized that defects created in our work were not enough in number to observe such a decrease in thermal stability, probably ascribed to the presence of extensive defects in the cited report (up to 1830 m^2^·g^–1^ BET surface area) instead quasi-punctual defects as reported here (970 m^2^·g^–1^) vs. the crystallographic value of 876 m^2^·g^–1^.^53^ Recently, some authors have demonstrated that symmetrically distributed (spatially correlated) defects in UiO-66 considerably improved the thermal properties compared to randomly distributed missing linkers,^54^ which is the most common situation. Defect arrangement induced by microwave radiation could be behind this phenomenon, as a consequence of dipolar moment alignment of modulator molecules in predefined directions when attaching the metal clusters generating this anisotropy, as previously described for other semiconductor materials.^55^ Demonstration, in addition to being complicated through advanced techniques (nanodomain crystalline resolution), is far from the scope of this work. This manner of generating defects favored the stability of the network and the cation impregnation in further steps and revealed to be crucial for potential utilization at the industrial level.^56^

ATR-FTIR spectra (Figure S4) showed the vibration corresponding to Zr–O (648 and 655 cm^–1^) probing the coordination between the ligand and the metal cluster after 5 and 20 min. COO vibrations, originally located at 1220 and 1670 cm^–1^, suffered a significant shift upon linker coordination to 1250–1253 and 1651–1655 cm^–1^, respectively, as well as a decrease in their intensity. Other minor differences between MOFs synthesized at different reaction times was the band at 780 cm^–1^ assigned to CCOO that disappeared in the spectra after 20 min but still remarkable after 5 min, demonstrating the high number of degrees of freedom still available in the structure and supporting the previous evidence, which highlight the gel behavior of such polymers.^57^

From a practical point of view, those MOFs obtained after 5 min were directly discarded because of the complexity in their handling (washing, centrifuging, etc.). Besides, we required the optimal compromise between defect concentration and other related physicochemical properties. The fact of showing up to 30% missing linkers, despite favoring mass transfer, would have negatively impacted the Pd loading because of the significant absence of –NH_2_ moieties. TGA curves (Figure S2) give a frank account on this extent, as materials obtained after 5 min reaction time showed a double jump in the region of 300–500 °C corresponding to both the linker and MOF decomposition. The optimal candidate selected for further analysis and Pd loading was that indicated in entry 10, Table S1.

With these aspects in mind, we extended this optimized protocol to a larger scale inside a multimodal microwave reactor. We were able to obtain up to 317 mg of material in a monomodal microwave (20 min of microwave-assisted synthesis vs. 24 h in the solvothermal method), while using a multimodal microwave, the synthesis was scaled up, producing ca. 1 g of material in only 20 min. Although *a priori* yields were lower using the multimodal reactor (91 vs. 84% in mono- and multimodal microwave instruments, respectively, *n* = 5), space-time yield (STY) values were higher in the large-scale procedure (1450 kg·m^–3^·day^–1^) because of the higher concentration of the chemical species compared to the former scenario (760 kg·m^–3^·day^–1^). Notably, physicochemical properties did not significantly differ between materials obtained using mono- and multimodal instruments, as deduced from the following section. Note that despite those materials obtained after 5 min were discarded for catalytic purposes (because of its poor crystallinity), we confirmed the positive match with the UiO-66 structure (Figure S1a), providing STYs > 3100 kg·m^–3^·day^–1^. This value is of extreme relevance for industrial fabrication as the highest value reported to date for this MOF in a continuous process was 2250 kg·m^–3^·day^–1^, indicating the suitability of this process for industrial scale-up.^58^ Finally, for catalytic purposes, UiO-66-NH_2_ nanocrystals were decorated with the active Pd species using a simple and efficient microwave-assisted reducing procedure (150 °C for 10 min), resulting in composites denoted as Pd(4.7%)@UiO-66-NH_2_ and Pd(1.7%)@UiO-66-NH_2_, containing 4.7 and 1.7 wt % Pd, respectively (based on ICP analysis).

### 3.2 Characterization of UiO-66-NH_2_ and Pd@UiO-66-NH_2_

PXRD patterns of pristine UiO-66-NH_2_, Pd(4.7%)@UiO-66-NH_2_, and Pd(1.7%)@UiO-66-NH_2_ (Figure S5) were compared to that corresponding to the simulated UiO-66 pattern (CCDC-1405751). Despite the success in the synthesis of this MOF, the peak broadening effect observed after Pd(II) loading and its further reduction to Pd NPs indicated a slight matrix degradation. It is worth mentioning that MOF matrices can be partially damaged when *in situ* preparing metal NPs under harsh reduction conditions (i.e., NaBH_4_). The absence of the characteristic diffraction peaks of Pd, e.g., the most intense at ca. 40° corresponding to the (111) plane, suggested the generation of nonaggregated Pd colloids with particle size smaller than the diffraction limit.^59^ The FTIR spectra of UiO-66-NH_2_ and Pd@UiO-66-NH_2_ composites were similar to those reported in the literature (Figure S6),^60^ including the most relevant bands: those of amino groups (NH_2_, 3500–3390 cm^–1^), carboxylic groups (COO, 1573 and 1385 cm^–1^), and aromatic rings (C=C, 1499 cm^–1^). Note that after the Pd incorporation, the IR bands were not significantly modified, probably due to the low Pd content. The thermal stability of the MOF and Pd composites was roughly estimated by thermogravimetric analysis (TGA) under an inert (Ar) atmosphere. In the TGA of UiO-66-NH_2_ (see Figure S7), three steps were observed: the first one corresponds to the water evaporation (100 °C), the second one is related to the weight loss due to the hydroxylation of the −OH groups (ca. 300–350 °C), and the last one (350 °C) is associated with the MOF decomposition and oxidation to ZrO_2_. Upon the Pd NP incorporation, the thermal stability of the Pd@UiO-66-NH_2_ composites decreases until 300 °C, which may be due to the generation of extended defects caused by microwave irradiation (see Figure S7), as described elsewhere.^27^ However, note here that the accurate estimation of the defect amount was not possible due to the lower material stability (i.e., defect concentration estimated as in ref (31) upon normalization of TGA curves at 600 °C, using 350 °C as the starting point of defect removal).

Further, in order to provide some insights into the dimension, distribution, and location of Pd NPs, gas sorption and HAADF–STEM characterization were performed on the UiO-66-NH_2_ and Pd@UiO-66-NH_2_ composites. The UiO-66-NH_2_ material showed the traditional type-I sorption isotherm (Figure S8), with a BET surface area (*S*_BET_) and micropore volume (*V*_p_) of 1035 m^2^·g^–1^ and 0.34 cm^3^·g^–1^, respectively, in agreement with reported values.^53^ After the Pd deposition, these values slightly decreased for Pd(1.7%)@UiO-66-NH_2_ (*S*_BET_ = 1001 m^2^·g^–1^ and V_p_ = 0.32 cm^3^·g^–1^) and more noticeably in the case of Pd(4.7%)@UiO-66-NH_2_ (*S*_BET_ = 757 m^2^·g^–1^ and *V*_p_ = 0.23 cm^3^·g^–1^), supporting the presence of Pd NPs within the MOF matrix.

**Figure 2:**
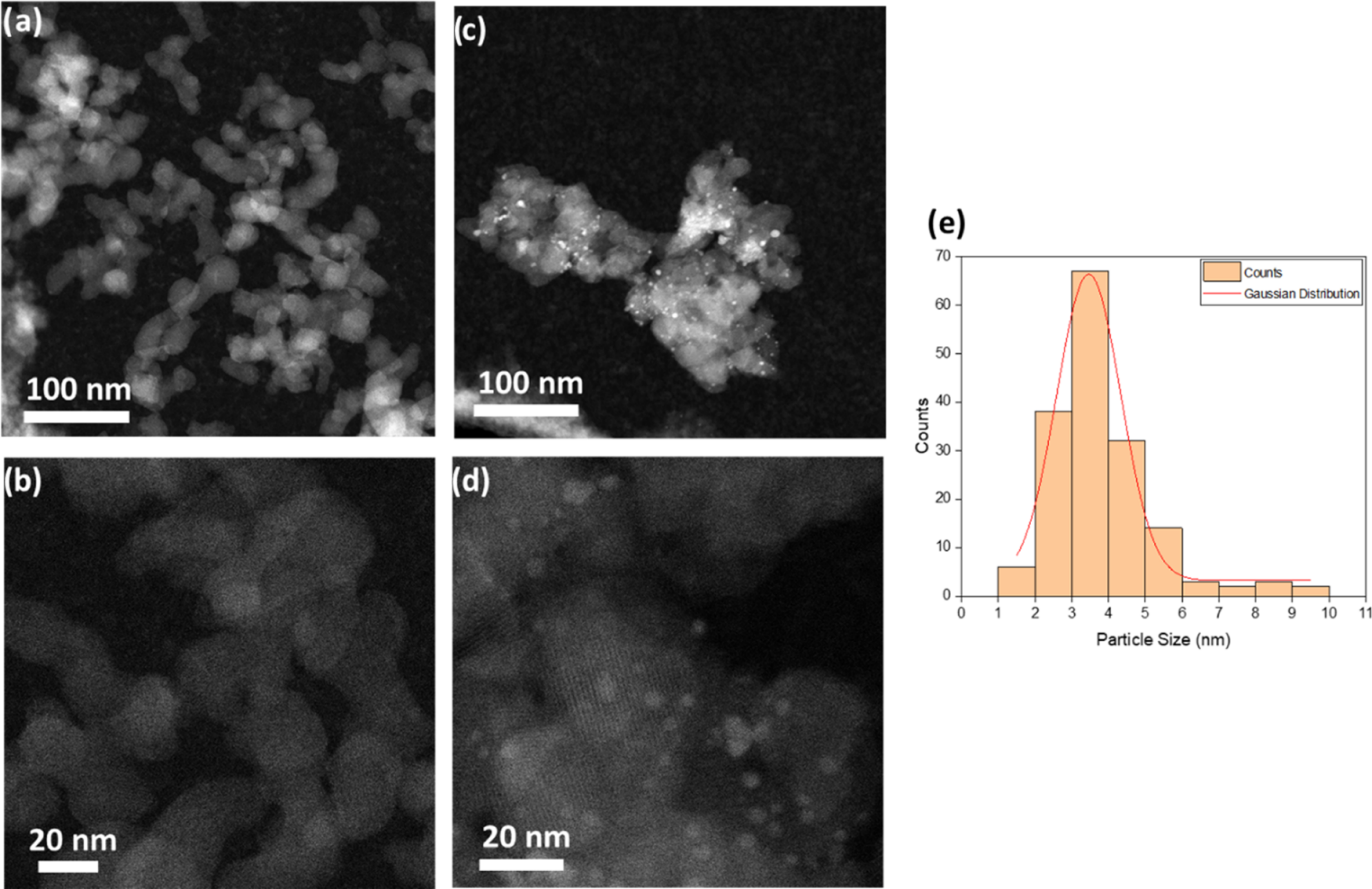
HAADF-STEM images of UiO-66-NH_2_ (a, b) and Pd(1.7%)@UiO-66-NH_2_ (c, d); magnification ×50,000 and ×150,000, respectively, and (e) particle size distribution of Pd(1.7%)@UiO-66-NH_2_.

In an attempt to precisely locate the Pd NPs, HAADF-STEM was performed for UiO-66-NH_2_ and Pd(1.7%)@UiO66-NH_2_. The morphology of the UiO-66-NH_2_ particles was preserved and remained unaltered after Pd(II) reduction under microwave radiation, as deduced from HAADF-STEM observations (Figure 2). The Pd NPs, with an estimated particle size of 3–4 nm (see Figure 2e), seem to be homogeneously distributed within the MOF matrix (Figure 2c,d, see Video 1).

### 3.3 Hydrogenation of Olefins

The catalytic activity of the Pd@UiO-66-NH_2_ composites was evaluated for the selective hydrogenation of olefins to the corresponding alkanes under mild conditions (1 bar H_2_, 25 °C). The reduction of 1-hexene was selected as a model reaction, comparing the catalytic activity of the composites (Pd@UiO-66-NH_2_) with several references: the commercial catalyst Pd/Al_2_O_3_, commonly used in this reaction,^61^ the pristine UiO-66-NH_2_, and PVP-stabilized Pd NPs (PVP-Pd NPs). Hexane (compound 2a) and 2-hexene (compound 3a) can be obtained as reduction and side isomerization products, respectively. Table 1 shows the results in terms of conversion of 1-hexene, its corresponding turnover frequency (TOF), and selectivity and yield to the targeted product (*n*-hexane) for the catalysts prepared and tested under different conditions.

**Table 1:**
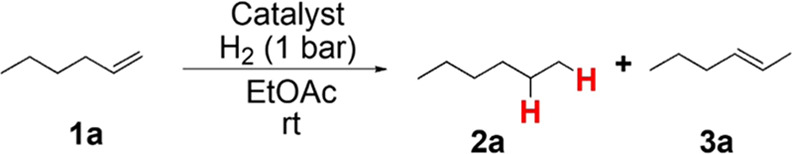
Catalytic Performance of the Different Samples in Terms of Conversion, Selectivity, Yield, and Turnover Frequency (TOF) Values^a^

entr y	catalyst^b^	mol %	time (h)	conversion (%)^e^	selectivity (%)^e^ (2a/3a)	yield (%)^e^ (2a)	TOF^f^ (h^–1^)
1	no catalyst		3	0	–/–	0	0
2	UiO-66-NH_2_		3	0	–/–	0	0
3	Pd(4.7%)@UiO-66-NH_2_	0.09	3	100	100/-	>99	377
4	Pd(1.7%)@UiO-66-NH_2_	0.03	3	100	100/-	>99	1043
5	Pd(1.7%)@UiO-66-NH_2_	0.03	2	100	100/-	>99	1565
**6**	**Pd(1.7%)**@UiO-66-NH_**2**_	**0.03**	**1**	**100**	**100/-**	>**99**	**3130**
7	Pd(1.7%)@UiO-66-NH_2_	0.03	0.5	72	63/47	56	
8	Pd(5%)/Al_2_O_3_^c^	0.03	1	100	82/18	70	2191
9	Pd(1.7%)@UiO-66-NH_2_^d^	0.03	6	100	100/-	94	7846
10	PVP-Pd NPs	0.05	1	6	42/58	3	
11	PVP-Pd NPs	0.10	1	100	46/54	45	393

a Reaction conditions: 10 mg of catalyst, 25 mL of a solution of 1-hexene (5 mmol) in ethyl acetate, 1 bar H_2_, and 25 °C, unless stated otherwise.

b wt % of Pd calculated by ICP-OES.

c Reaction conditions: 3.4 mg of catalyst and 25 mL of a solution of 1-hexene (5 mmol) in ethyl acetate.

d Reaction under solvent-free conditions: 10 mg of catalyst, 10 mL of 1-hexene (80 mmol), 8 bar H_2_, and 25 °C for 6 h.

e Conversion, selectivity, and yield were determined by GC using cyclohexanol as an internal standard and the corresponding products were identified by GC–MS.

f TOF calculated as mol of product (hexane) per mol of metal per hour.

Reference reactions (entries 1 and 2), either in the absence or presence of the parent MOF, resulted in nonmeasurable conversion, confirming that the Pd species are mainly responsible for the catalytic activity. Subsequently, the catalytic performance of the Pd(4.7%)@UiO-66-NH_2_ composite, with a similar Pd content as that found in the commercial catalyst (5%), was evaluated. After 3 h, a total conversion with excellent selectivity toward hexane was achieved (entry 3). Interestingly, when the Pd loading of the composite decreased from 4.7 to 1.7%, similar catalytic results were reached (100% conversion and 100% selectivity to hexane), highlighting the outstanding catalytic activity of the defective Pd(1.7%)@UiO-66-NH_2_ (entry 4), with remarkably higher TOF values than those obtained with a higher Pd loading.

Reaction time was then shortened to optimize the catalytic conditions, observing full conversion and 100% selectivity toward hexane after 2 and 1 h (entries 5 and 6), achieving an excellent TOF value (3130 h^–1^, entry 6). Nevertheless, these values significantly decreased (72% conversion and 63% selectivity) when the reaction proceeded for only 0.5 h (entry 7). Thus, 1 h was selected as the optimal reaction time. The competition between isomerization and reduction has been reported before for this reaction.^62^ Previous studies using MOF-based composites did not usually observe this effect because of the used extreme reaction conditions (high H_2_ pressure and/or long reaction times, which favor the olefin hydrogenation) unlike the milder conditions in this work (1 bar H_2_).

**Figure 3:**
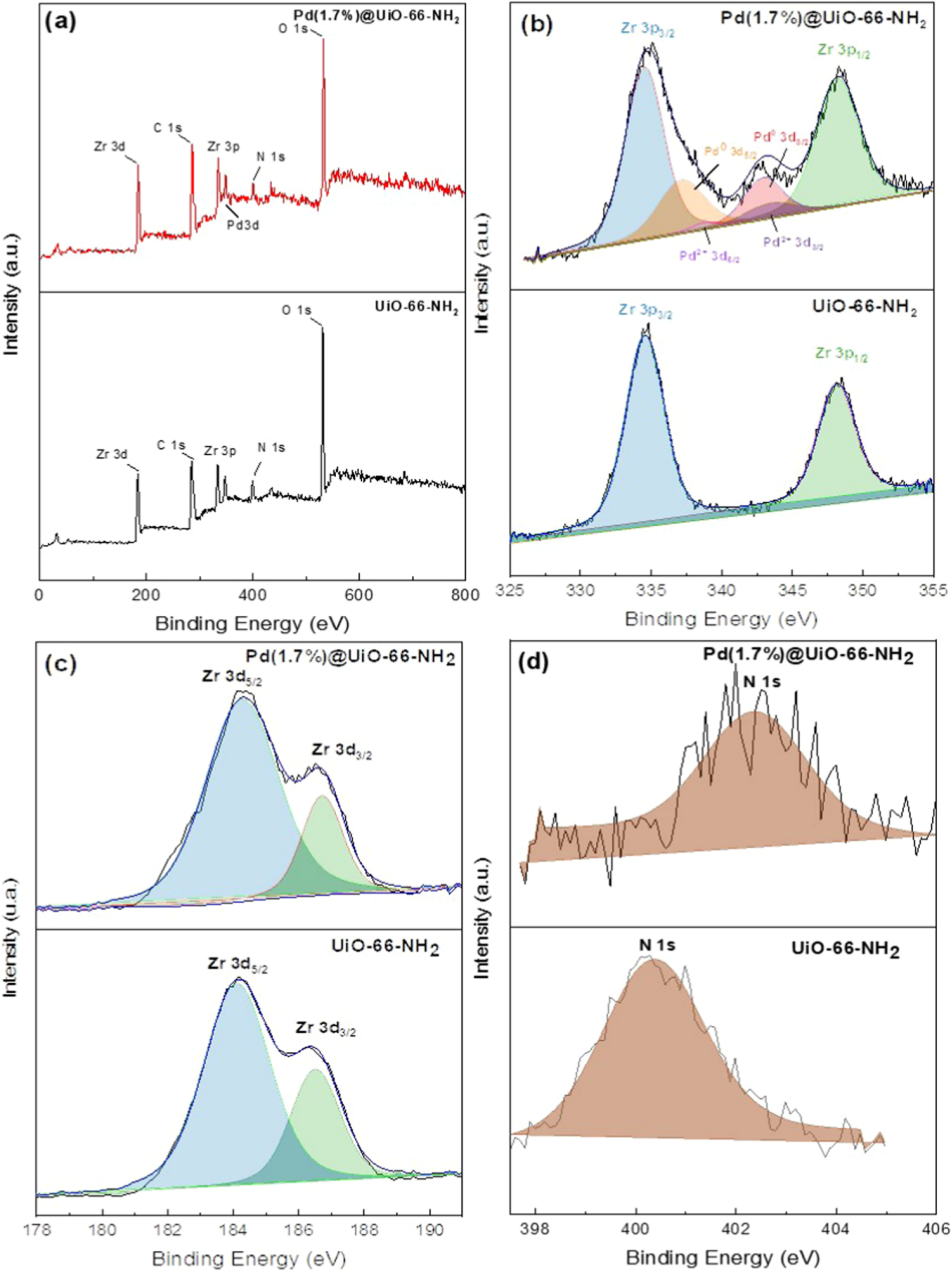
XPS spectra of UiO66-NH_2_ and Pd(1.7%)@UiO-66-NH_2_ (a) survey, (b) Zr 3p and Pd 3d regions, (c) Zr 3d region, and (d) N 1s region.

Pd leaching was discarded under such conditions, an aspect that was confirmed when removing Pd(1.7%)@UiO-66-NH_2_ from the media after 15 min reaction time (51% conversion). Then, the resulting supernatant was stirred for extra 45 min in the absence of the catalyst. Under such conditions, conversion of 1-hexene did not increase (Figure S9), suggesting the existence of a strong interaction between the Pd NPs and the MOF, avoiding their release to the media, as conversion would have increased otherwise. In addition, the oxidation states of the constituent elements of the UiO-66-NH_2_ and Pd(1.7%)@UiO-66-NH_2_ catalysts were analyzed by X-ray photoelectron spectroscopy (XPS). XPS spectral survey (Figure 3a) shows the presence of Zr, C, O, and N atoms in both samples, which are the main components of the MOF. The 3d Pd signal is only observed in Pd(1.7%)@UiO-66-NH_2_, which can be deconvoluted to four peaks (Figure 3b). The strong Pd 3d_5/2_ and Pd 3d_3/2_ peaks at 337 and 342 eV, respectively, are attributed to Pd^0^, confirming the formation of Pd NPs. Nevertheless, the two less intense peaks at 338 and 343 eV can be assigned to Pd^2+^,^63^ suggesting that a small fraction of Pd remains oxidized in the composite. This can be due to the reoxidation of the dispersed Pd NPs in the presence of air, which agrees with previous studies.^64^ In both spectra, the Zr 3p doublet is observed at ca. 334 and 348 eV (Figure 3b), while the Zr 3d signal can be deconvoluted into two peaks (184 and 186 eV), assigned to Zr^4+^ (Figure 3c). Finally, the N 1s peak for Pd(1.7%)@UiO-66-NH_2_ is slightly shifted to higher binding energy values (402 eV) and broadened as compared to the spectrum of UiO-66-NH_2_ (400 eV) (Figure 3d), suggesting the formation of interactions between the amine groups of the ligand of the MOF and the Pd species.^35^

Upon the catalytic reaction, Pd(1.7%)@UiO-66-NH_2_ was analyzed by PXRD and XPS. The crystalline structure of the MOF-based composite was kept (see Figure S10), and the Pd 3d XPS spectrum (Figure S11) showed only the peaks corresponding to Pd^0^ (337 and 342 eV), which suggests that the reoxidized Pd^2+^ species, present in the fresh composite catalyst, do not exist after the catalytic hydrogenation (1 h reaction time). Note that the reoxidized Pd^2+^ species could persist after 30 min reaction time, regardless of the presence of H_2_ gas. This could explain the lower selectivity (63% selectivity, entry 7), since Pd(II) catalyzes isomerization vs. hydrogenation.^65^ In addition, ICP analyses confirmed that the Pd content before and after catalysis remains the same (1.69 ± 0.08 vs. 1.56 ± 0.08%), supporting the absence of Pd leaching during the reaction.

The performance of the composite was compared to that of a commercially available catalyst, Pd(5%)@Al_2_O_3_. In this case, full conversion was also achieved, but the selectivity toward the alkane was lower (82% selectivity, entry 8), which could be ascribed to the presence of Pd(II) species.^66^ Besides, Pd(1.7%)@UiO-66-NH_2_ exhibited better hydrogenation activity than that achieved by the commercial catalyst (TOF = 3130 vs. 2191 h^–1^), suggesting a potential catalytic enhancement of Pd NPs when incorporated within the MOF matrix and despite the higher Pd loading in the latter material. Comparing the catalytic results of our composite with other reported studies (Table S2), the catalytic hydrogenation activity of Pd(1.7%)@UiO-66-NH_2_ was significantly superior to that observed with other UiO-66-based composites (e.g., the hierarchically porous Pt@UiO-66-NH_2_-2h:^67^ TOF = 1007 h^–1^ or Pt@UiO-66:^68^ TOF = 500 h^–1^) and other well-known MOF composites (Pt/MIL-88b (Cr):^69^ TOF = 1052 h^–1^ or Pt/NiBDC (Ni):^69^ TOF = 989 h^–1^).

In order to shed some light on the effect of the MOF as a Pd NP host, the hydrogenation of 1-hexene was carried out in the presence of PVP-Pd NPs, which exhibit a similar average size compared to Pd NPs in the UiO-66-NH_2_ (average size of 4 nm, see Figure S12). The tested catalytic content was higher than that in the composite (entries 10 and 11, 0.05 and 0.10 vs. 0.03 mol %). Lower conversion (6%, entry 10) was obtained when using 0.05 mol %, supporting the role of the MOF as a synergistic catalytic platform for well-accessible and dispersed Pd NPs. Only after using 0.10 mol % PVP-Pd NPs (entry 11), complete conversion was reached, but a quite lower selectivity was found in both cases (42–46% selectivity toward alkane), stressing the relevance of the support in this process. In addition, the reaction was also performed over the Pd(1.7%)@UiO66-NH_2_ composite under solvent-free conditions. This provides a safer and more environmentally friendly process for the efficient production of alkanes from alkenes. Remarkably, Pd(1.7%)@UiO66-NH_2_ successfully catalyzed the solvent-free hydrogenation of 1-hexene (10 mL, 0.08 mol) into the corresponding hexane, after 6 h and only consuming 8 bar H_2_, demonstrating its high catalytic hydrogenation performance in solvent-free mode (TOF = 7846 h^–1^).

A screening of different olefins as substrates was performed under the optimized conditions, with the results being presented in Table 2. In the case of linear alkenes (1-octene and 1-tridecene, entries 1 and 2, respectively), the length of the alkyl chain had a negligible impact on the catalytic performance (full conversion, >95% yield). Similar results were observed in the hydrogenation of cyclohexene (entry 3) and anethole (entry 4). The generation of byproducts was negligible in the reactions mentioned above, demonstrating that Pd(1.7%)@UiO-66-NH_2_ shows a superior selectivity toward the alkane formation. Finally, the hydrogenation of a bulky alkene, tetraphenyl ethylene, was also investigated (entry 5). The dimensions of this alkene (6.7 Å) may hinder its diffusion throughout the cavities of UiO-66-NH_2_ (6.0 Å).^36^ Thus, the composite showed a relatively low conversion (32%, entry 5), supporting the presence of some catalytically active species on the outer surface of the composite. However, the main active Pd species, also responsible for the selectivity, might be confined inside the MOF matrix, in agreement with the HAADF-STEM observations (Figure S13 a–c).

**Table 2:**
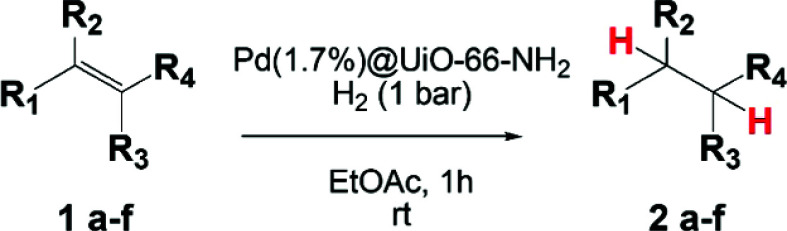

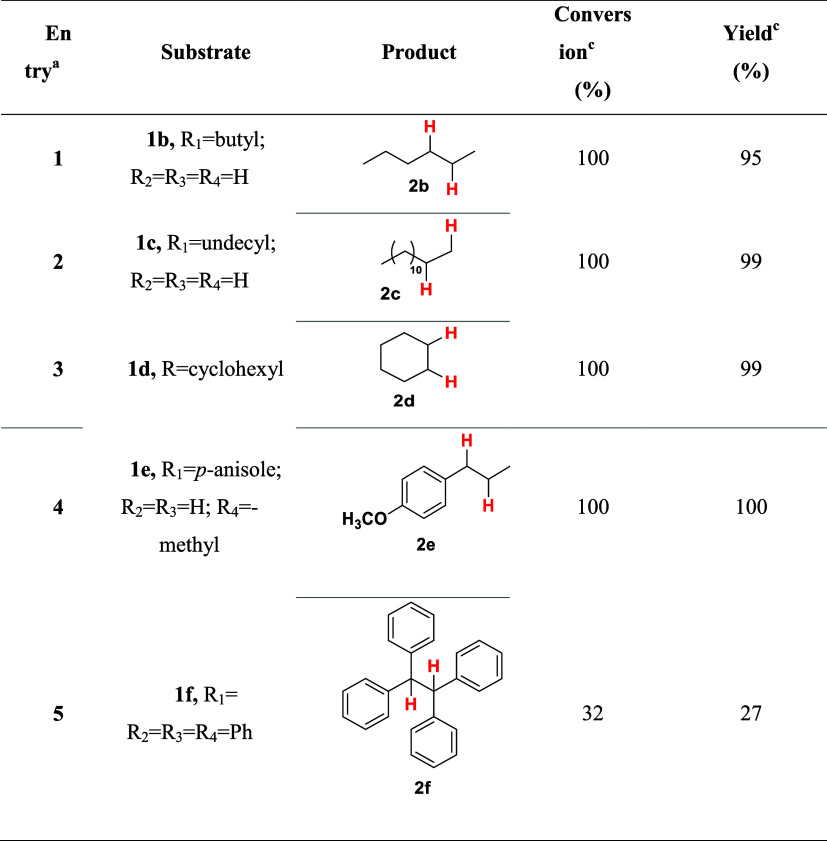
Reaction Scope^a^

a Reaction conditions: 10 mg of Pd(1.7%)@UiO-66-NH_2_, 25 mL of a solution of alkene (5 mmol) in ethyl acetate, 1 bar H_2_, and 25 °C. Conversion and yield were determined by GC, and the corresponding products were identified by GC–MS using cyclohexanol as an internal standard.

**Scheme 1:**
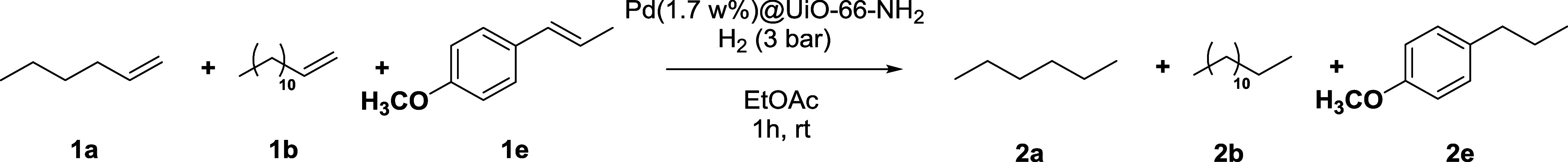
Hydrogenation of a Complex Mixture of Alkenes using Pd(1.7%)@UiO-66-NH_2_

To further probe the catalytic hydrogenation properties of the Pd(1.7%)@UiO-66-NH_2_ platform under mild conditions (3 bar H_2_, 25 °C, 1 h), a complex mixture of alkenes (including 1-hexene, 1-tridecene, and anethole) was tested (Scheme 1). The conversion and alkane selectivity for the three alkenes were 100%, showing no preference for any substrate.

**Figure 4:**
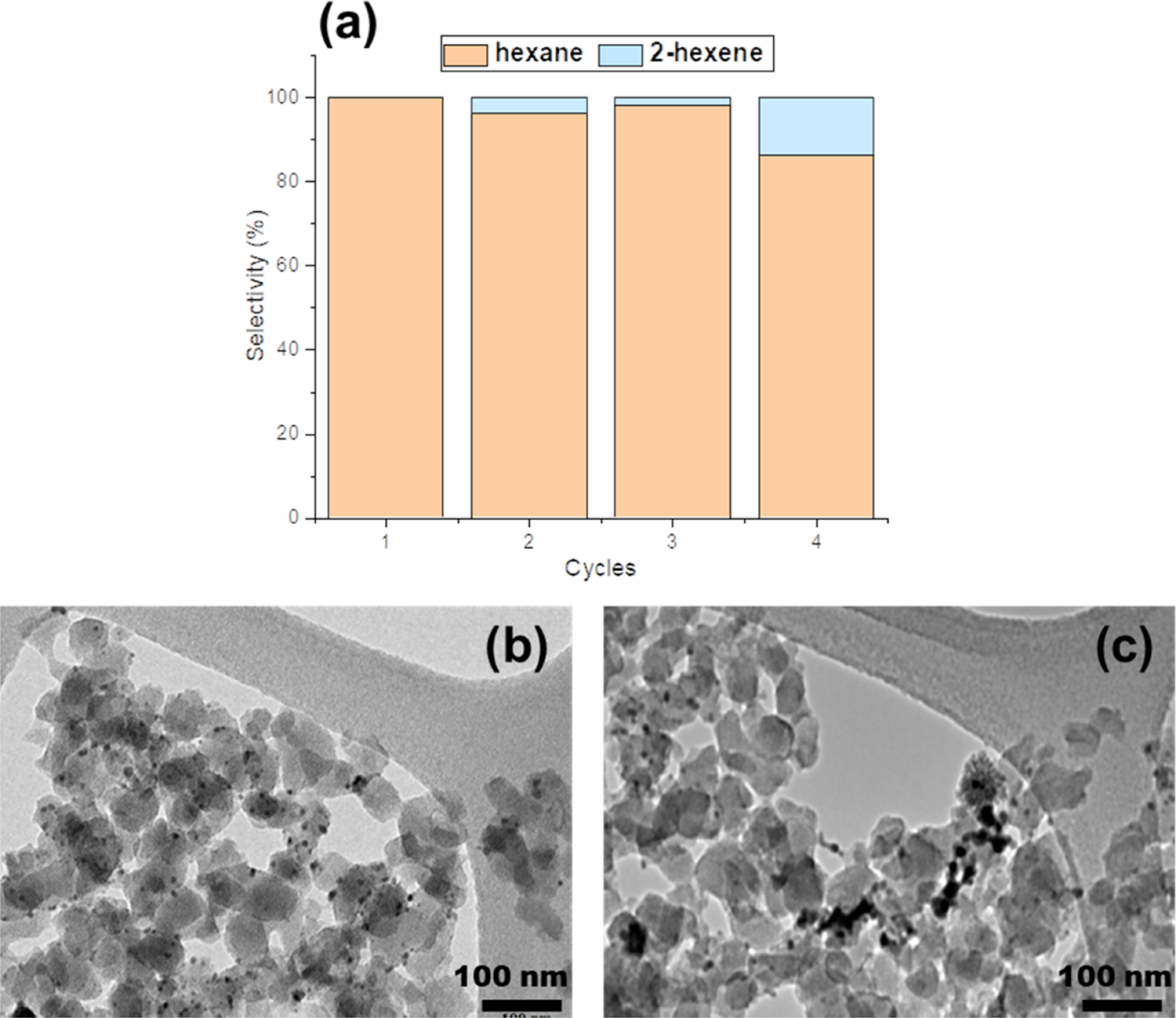
(a) Hydrogenation cycles of the Pd(1.7%)@UiO-66-NH_2_ catalyst and TEM images of (b) fresh Pd(1.7%)@UiO-66-NH_2_ catalyst and (c) Pd(1.7%)@UiO-66-NH_2_ catalyst after four cycles.

The recyclability of the Pd(1.7%)@UiO-66-NH_2_ catalyst was studied for the hydrogenation of 1-hexene without applying any regeneration treatment. The results indicated that the high activity of the composite was preserved after four cycles, observing a slight decrease in the selectivity in the fourth run 100 vs. 86%. This might be caused by a Pd NP migration/aggregation (Figure 4c) and a decrease in the MOF crystallinity (Figure S14).

Precisely locating Pd NPs within/onto MOFs is extremely challenging both from the synthetic and characterization points of view. However, several pieces of evidence throughout this work have supported that catalytically active species are equally distributed within and onto UiO-66-NH_2_ crystals. For instance, the conversion of the bulkier tetraphenyl ethylene, which is not able to diffuse to CUSs inside the MOF, is also in agreement with the partial location of Pd NPs onto the MOF external surface. To preferentially remove the Pd NPs located on the outer MOF crystal while preserving those inside the porosity/framework, the composite Pd(1.7%)@UiO-66-NH_2_ was treated with a solution of K_2_S_2_O_8_ (5 equiv) in HCl (2 M) (Figure S15). This reagent was previously used for the oxidative redispersion of Pd(0) into Pd(II) cations in a different MOF, the MIL-101-NH_2_.^70^ Thus, this treatment successfully eliminated the external Pd NPs, keeping those inside the porosity/framework, as confirmed by the HAADF–STEM images (Figure S13d–f). Upon an oxidative treatment of only 15 min, the material continued demonstrating an important catalytic activity for olefin hydrogenation but was associated with a significant loss of selectivity (entry 2, Table 3). This aspect was exacerbated when the oxidative treatment lasts for 45 min (entry 3), showing not only a selectivity decrease (from 100 to 62%) but also a reduction in the yield (from 100 to 53%). Note here that ICP revealed not significant differences on the Pd content, regardless of the oxidation extent (entries 2 and 3). The loss of selectivity was then associated with the presence of Pd(II) species, as mentioned above. Overall, Pd NPs are then associated with the reduction reaction, while isomerization is produced by the Pd(II) species. Further, the origin of the selectivity seems to reside in those Pd NPs located within the MOF porosity/framework, as demonstrated by those reactions performed with PVP-Pd NPs (entries 10 and 11; Table 1). Pd species inside the MOF porosity were eventually oxidized during consecutive catalytic runs, as observed in Figure 4, losing the overall selectivity as a consequence of their progressive oxidation into Pd(II).

**Table 3:** Hydrogenation of 1-Hexene Using Pd(1.7%)@UiO-66-NH_2_ before and after Oxidative Treatment

entry	catalyst	metal loading (wt %)	treatment	yield (%)	selectivity (2a/3a)
1	Pd@UiO-66-NH_2_	1.74 ± 0.08	-	100	100/-
2	Pd@UiO-66-NH_2_ (ox-15)	1.63 ± 0.08	K_2_S_2_O_8_/HCl (15 min)	100	95/5
3	Pd@UiO-66-NH_2_ (ox-45)	1.60 ± 0.08	K_2_S_2_O_8_/HCl (45 min)	53	62/38

## 4 Conclusions

In this work, we have optimized the synthesis of defective UiO-66-NH_2_ nanocrystals (91% yield on a metal basis; 760 kg·m^–3^·day^–1^) for improving their stability and catalytic performance. The optimal candidate, obtained in a monomodal microwave reactor, showed a compromise between defects (18.2%, as missing linkers), textural properties (937 m^2^·g^–1^ BET surface area), thermal stability (350 °C decomposition temperature by TGA), and particle size (23.4 nm). The procedure was successfully scaled up using a multimodal microwave reactor, highlighting the industrial relevance of this method (86% yield metal basis; 1450 kg·m^–3^·day^–1^). The resulting MOF was loaded with Pd(II) through wet impregnation followed by microwave radiation (150 °C, 10 min) to obtain small and homogeneously distributed Pd NPs (3–4 nm), as demonstrated by HAADF-STEM. The defective Pd(1.7%)@UiO-66-NH_2_ composite was able to selectively hydrogenate challenging alkenes under milder reaction conditions (full conversion by using 1 bar H_2_, room temperature) than those used in previous studies. We identified the robust interaction and synergistic effects between both materials (Pd NPs and UiO-66-NH_2_) in the composite as the origin of the selectivity (hydrogenation vs. isomerization), achieving superior activity compared to the commercially available Pd(5%)/Al_2_O_3_ catalyst (TOF = 3130 vs. 2191 h^–1^, respectively), with recyclability for three consecutive runs.

## Supporting Information Available

Additional experimental details, PXRD patterns, TGA curves, nitrogen sorption isotherms, FTIR spectra, leaching test, XPS spectra, TEM image, HAADF-STEM images, and ^1^H NMR spectra of catalytic products, and comparison of catalytic hydrogenation activity of MOF-based composites (PDF)
Video 1 (MP4)
