# Supplementary material for: Scaling-Up Microwave-Assisted Synthesis of Highly Defective Pd@UiO-66-NH2 Catalysts for Selective Olefin Hydrogenation under Ambient Conditions
Source: ACS Appl Mater Interfaces. 2024 Apr 26;16(18):24108–21. doi: 10.1021/acsami.4c03106 (PMC11082845; doi:10.1021/acsami.4c03106)

Supporting Information

Scaling-up microwave-assisted synthesis of highly defective Pd@UiO-66-NH_2 catalysts for selective olefin hydrogenation under ambient conditions

Raúl M. Guerrero [a,b], Ignacio D. Lemir [a,b], Sergio Carrasco [a], Carlos Fernández-Ruiz [a,b], Safiyye Kavak [c], Patricia Pizarro [b,d], David P. Serrano [b,d], Sara Bals [c], Patricia Horcajada [a]*, Yolanda Pérez [a,e]*

[a] *Advanced Porous Materials Unit, IMDEA Energy Institute, Avda. Ramón de la Sagra, 3, Móstoles, 28935 Madrid, Spain*

[b] *Thermochemical Processes Unit, IMDEA Energy Institute, Avda. Ramón de la Sagra, 3, Móstoles, 28935 Madrid, Spain*

[c] *EMAT and NANOlab Center of Excellence, Univ. of Antwerp, Groenenborgerlaan 171, Antwerp 2020, Belgium*

[d] *Chemical and Environmental Engineering Group, Rey Juan Carlos University, C/ Tulipán, s/n, Móstoles, 28933 Madrid, Spain*

[e] *COMET-NANO Group, ESCET, Universidad Rey Juan Carlos, C/ Tulipán, s/n, Móstoles, 28933, Madrid, Spain*

*__Corresponding Author E-mail:__ yolanda.cortes@urjc.es (Y. Pérez); patricia.horcajada@imdea.org (P. Horcajada)

Table S1. Optimization of defective and nano-sized UiO-66-NH_2 obtained under microwave radiation. Syntheses conditions and some of their physicochemical properties obtained upon characterization.

Entry	Synthesis						Characterization					
	Modulator (mmol)		Precursor	Time (min)	MOF amount (mg)	P (bar)	Crystal domain size (nm)[a]	Hydrodynamic diameter (nm)[b]	MOF decomp. ($T_{1/2}$, ºC)[c]	Linker defects (%)[d]	S_{BET} (m^2/g)[e]	V_p (cm^3/g)[f]
1	TFA	5.23	$ZrOCl_2$	5	330.3	4.5	19.7	240(40)	462	21.3	815	0.15
2	TFA	9.21	$ZrOCl_2$	5	328	4.8	24.5	170(30)	470	24.2	876	0.19
3	TFA	13.07	$ZrOCl_2$	5	325.6	5.4	31.0	90(20)	473	29.1	971	0.26
4	TFA	13.07	$Zr(OPr)_4$	5	191.7	4.4	38.1	200(60)	469	32.9	796	0.26
5	AcOH	17.48	$ZrOCl_2$	5	315.3	4.8	15.5	260(30)	383	11.6	739	0.13
6	TfOH	11.33	$ZrOCl_2$	5	62.7	5.4	33.0	230(50)	476	4.4	499	0.17
7	TFA	0.00	$ZrOCl_2$	20	321.1	5.2	11.4	140(20)	375	0.2	585	0.06
8	TFA	3.27	$ZrOCl_2$	20	311.6	8.1	13.0	290(40)	419	9.0	590	0.07
9	TFA	6.53	$ZrOCl_2$	20	308.1	11.4	15.1	240(30)	451	12.8	626	0.10
10	TFA	9.21	$ZrOCl_2$	20	316.7	14.0	23.4	220(30)	467	18.2	937	0.22
11	TFA	9.21	$ZrCl_4$	20	161.5	17.5	15.1	320(50)	461	3.6	182	0.01
12	TFA	9.21	$Zr(OPr)_4$	20	351.1	13.3	33.6	270(50)	470	-0.9	867	0.03

Reactions were performed inside a 30-mL microwave vial at 175 ºC (heating ramp, 5 min), using 0.92 mmol Zr(IV) source, 0.92 mmol 2ATA, 10 mL DMF and the indicated amount of modulator. [a] Particle size estimated by PXRD using Scherrer equation from the peak of the (111) plane as in REF[1] . [b] Measured by DLS in EtOH. [c] Decomposition temperature ($T_{1/2}$) as the maximum of the first derivative of each TGA curve. [d] Defect concentration estimated as in REF[2] upon normalization of TGA curves at 600 ºC. [e] BET surface area estimated from nitrogen adsorption isotherm (branch p/p_0 = 0.04-0.20). [f] Pore volume from the t-plot using the Harkins and Jura method (branch p/p_0 = 0.25-0.60). Textural properties calculated using Tristar II Plus Version 3.03.

(1) Solís, R. R.; Peñas-Garzón, M.; Belver, C.; Rodriguez, J. J.; Bedia, J. Highly Stable UiO-66-NH2 by the Microwave-Assisted Synthesis for Solar Photocatalytic Water Treatment. *J. Environ. Chem. Eng.* **2022**, *10* (2), 107122.

(2) Yin, J.; Kang, Z.; Fu, Y.; Cao, W.; Wang, Y.; Guan, H.; Yin, Y.; Chen, B.; Yi, X.; Chen, W.; Shao, W.; Zhu, Y.; Zheng, A.; Wang, Q.; Kong, X. Molecular Identification and Quantification of Defect Sites in Metal-Organic Frameworks with NMR Probe Molecules. *Nat. Commun.* **2022**, *13* (1), 5112.

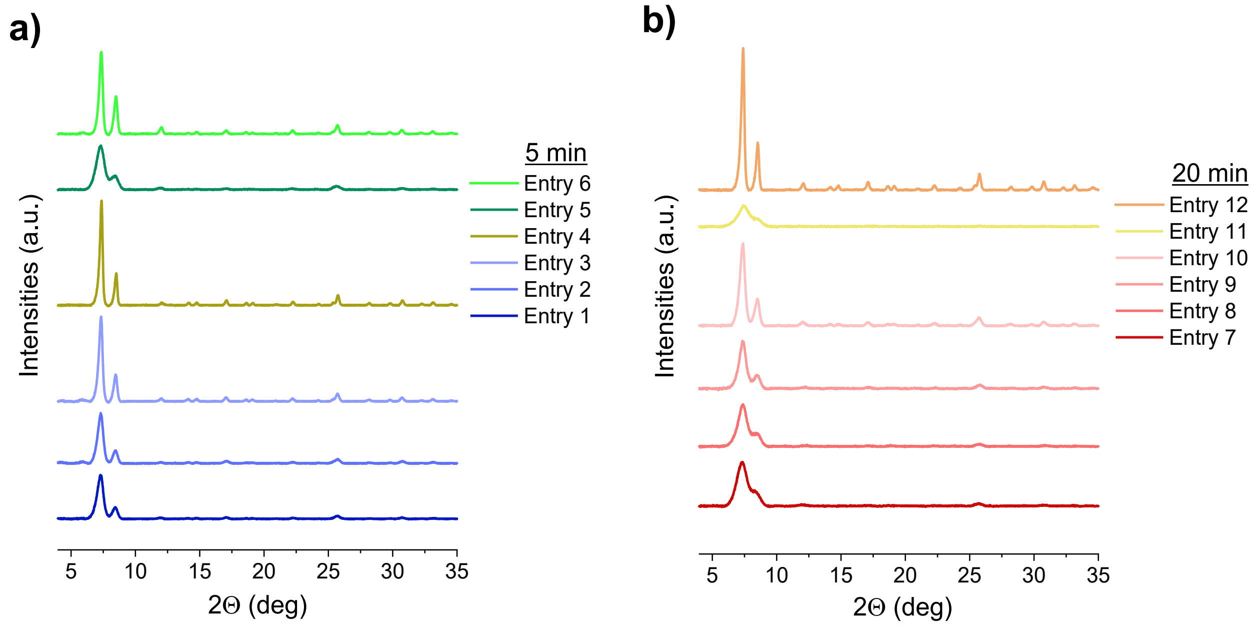

Figure S1. PXRD patterns of UiO-66-NH_2 materials obtained during microwave-assisted synthesis optimization after a) 5 min; and b) 20 min reaction time.

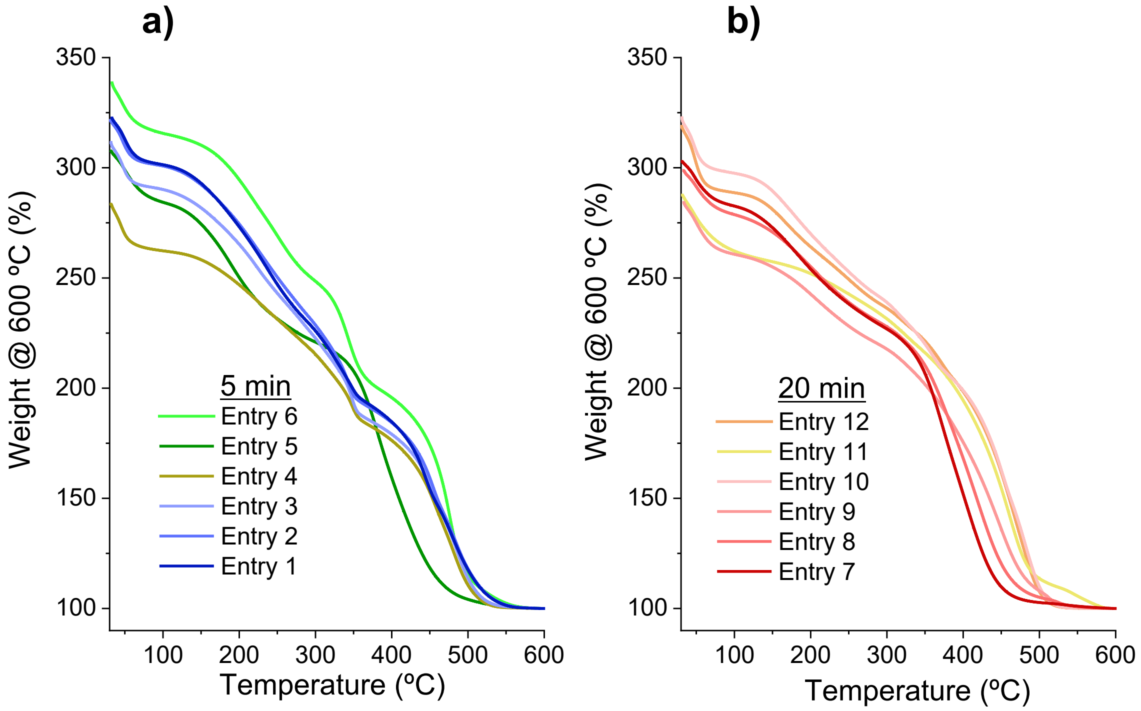

Figure S2. TGA curves of UiO-66-NH_2 materials obtained during microwave-assisted synthesis optimization after: a) 5 min; and b) 20 min reaction time. Normalization of TGA curves at 600 ºC for the estimation of defect concentration [2]

a)

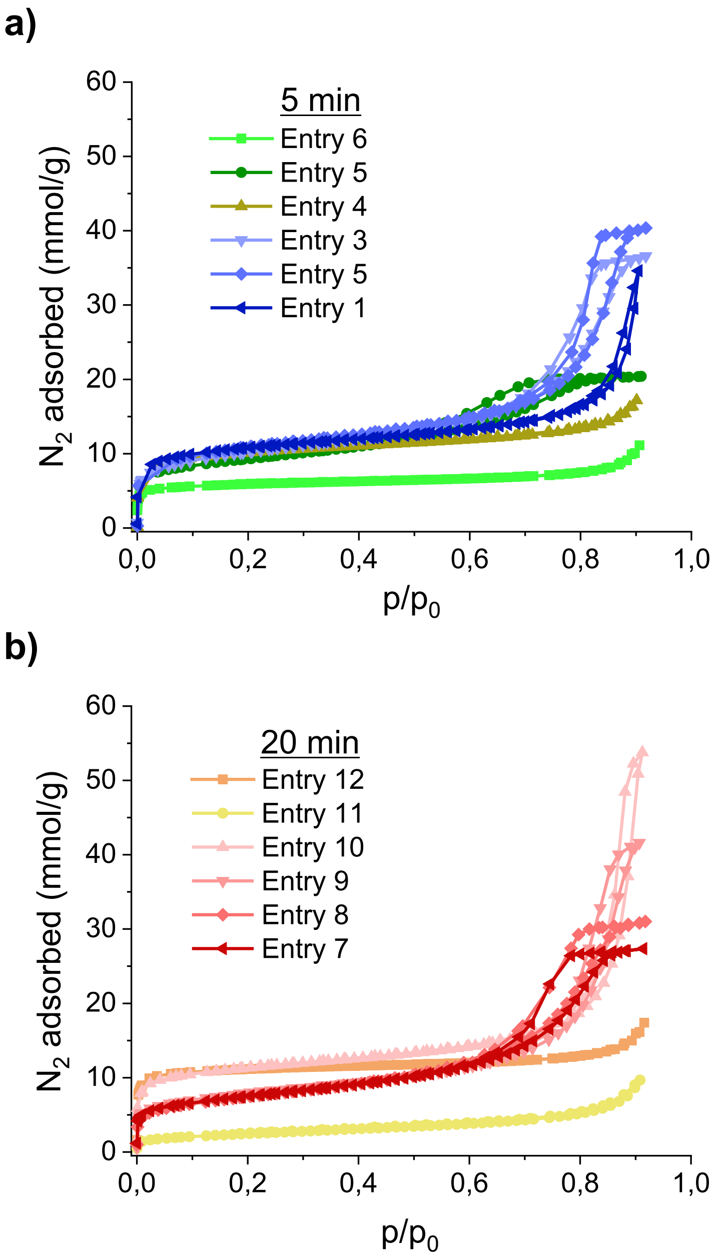

Figure S3. Nitrogen sorption isotherms at 77K of UiO-66-NH_2 materials obtained during microwave-assisted synthesis optimization after: a) 5 min; and b) 20 min reaction time.

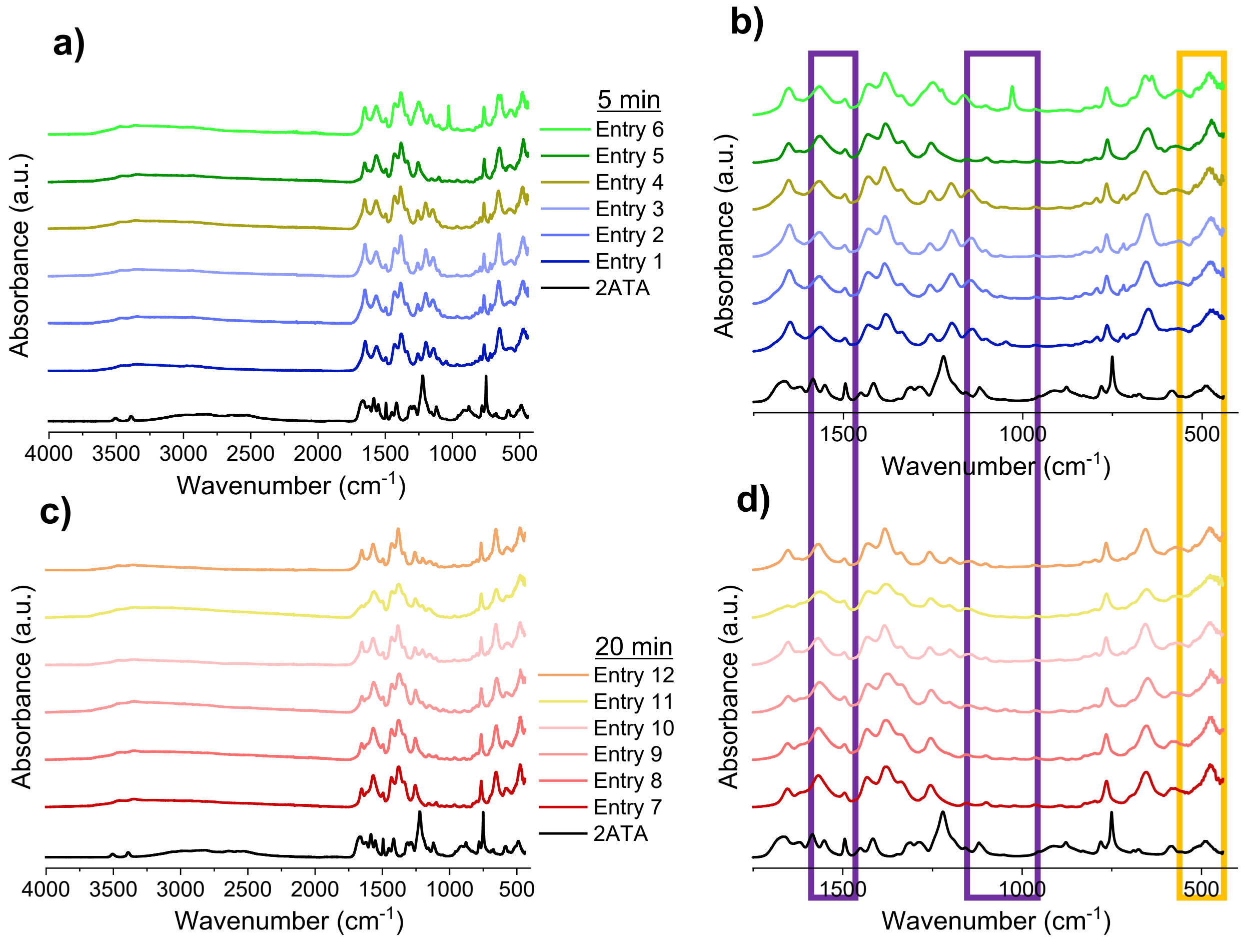

Figure S4. ATR-FTIR spectra of UiO-66-NH_2 materials obtained during microwave-assisted synthesis optimization after: a) 5 min; and c) 20 min reaction time. b) and d) correspond to their amplification within 400-1750 cm^{-1}. Purple frame: modification of C=O and C-O vibrations from the linker (2-aminoterephthalic acid, 2ATA) upon metal coordination in terms of shift and intensity. Yellow frame: the emergence of Zr-O vibration band, absent in 2ATA

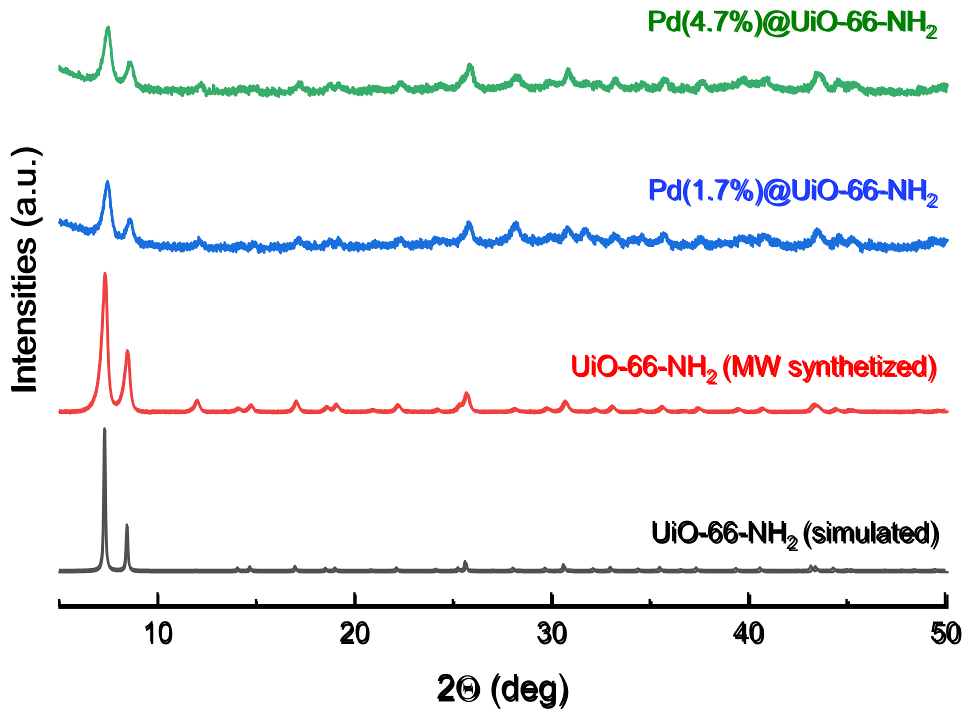

Figure S5. Simulated PXRD pattern of UiO-66-NH_2 (black) and experimental ones of UiO-66-NH_2 (red), Pd(1.7%)@UiO-66-NH_2 (blue) and Pd(4.7%)@UiO-66-NH_2 (green)

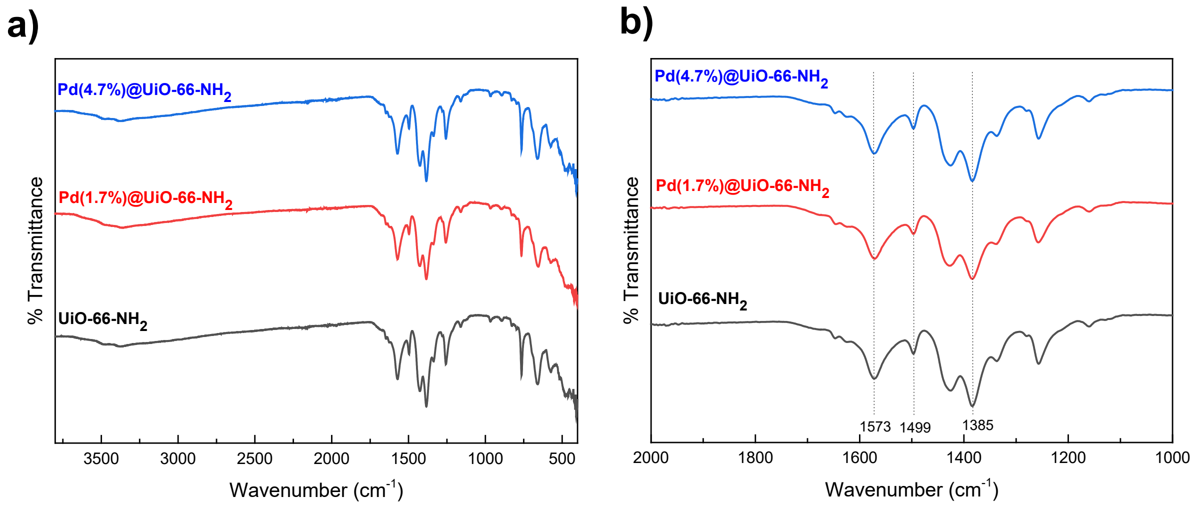

Figure S6. a) FTIR spectra of UiO-66-NH_2 (black), Pd(1.7%)@UiO-66-NH_2 (red) and Pd(4.7%)@UiO-66-NH_2 (blue). b) Zoom of the ATR-FTIR spectra in the range of 2000-1000 cm^{-1}

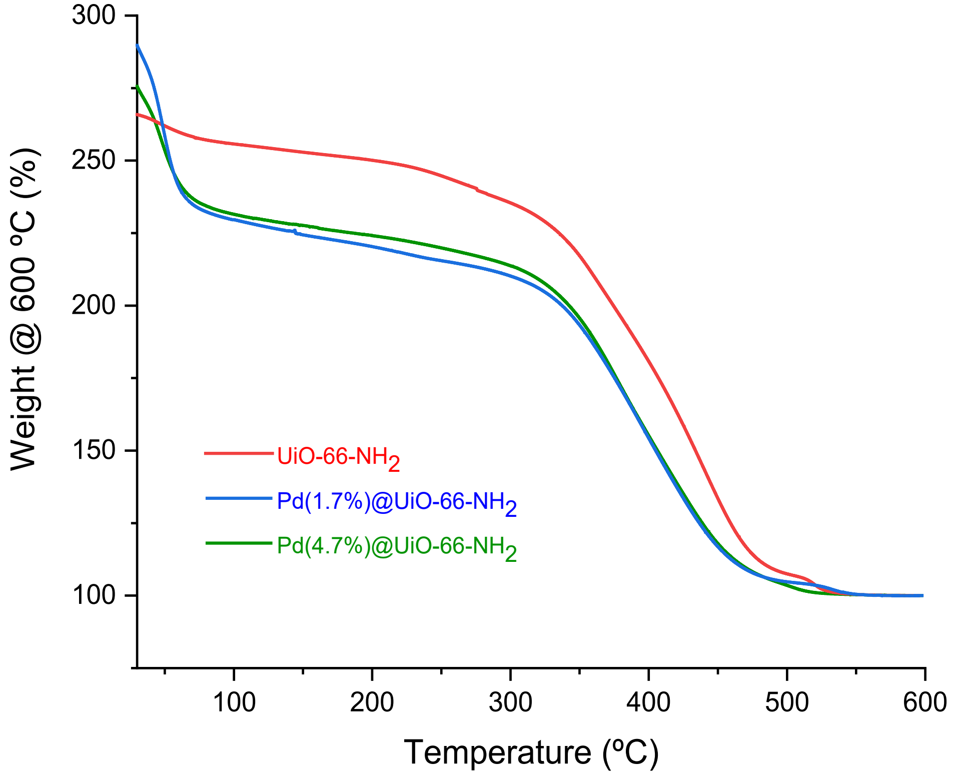

Figure S7. TGA curves of UiO-66-NH_2, Pd(1.7%)@UiO-66-NH_2 and Pd(4.7%)@UiO-66-NH_2. Normalization of TGA curves at 600 ºC for the estimation of defect concentration [2].

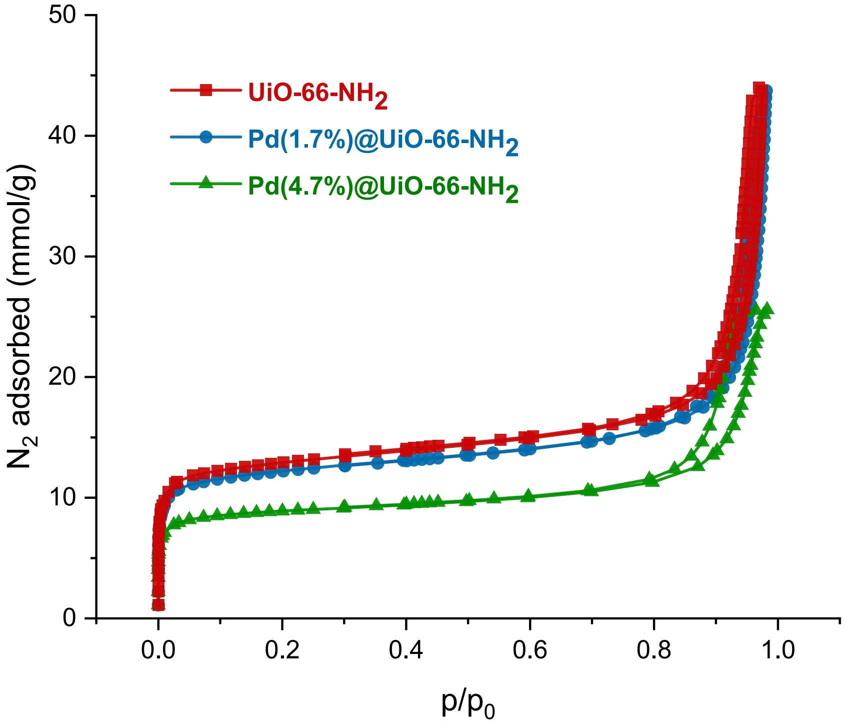

Figure S8. Nitrogen sorption isotherms at 77 K of UiO-66-NH_2 (red); Pd(1.7%)UiO-66-NH_2 (blue) and Pd(4.7%)@UiO-66-NH_2 (green).

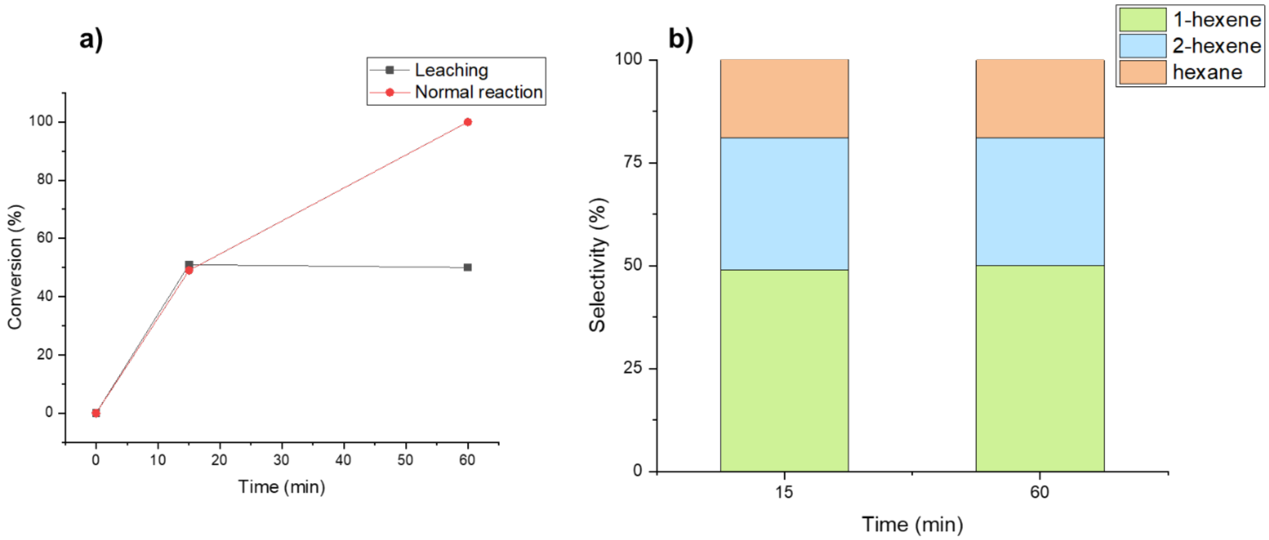

Figure S9. Leaching test: a) Conversion *vs*. time b) Selectivity *vs*. time.

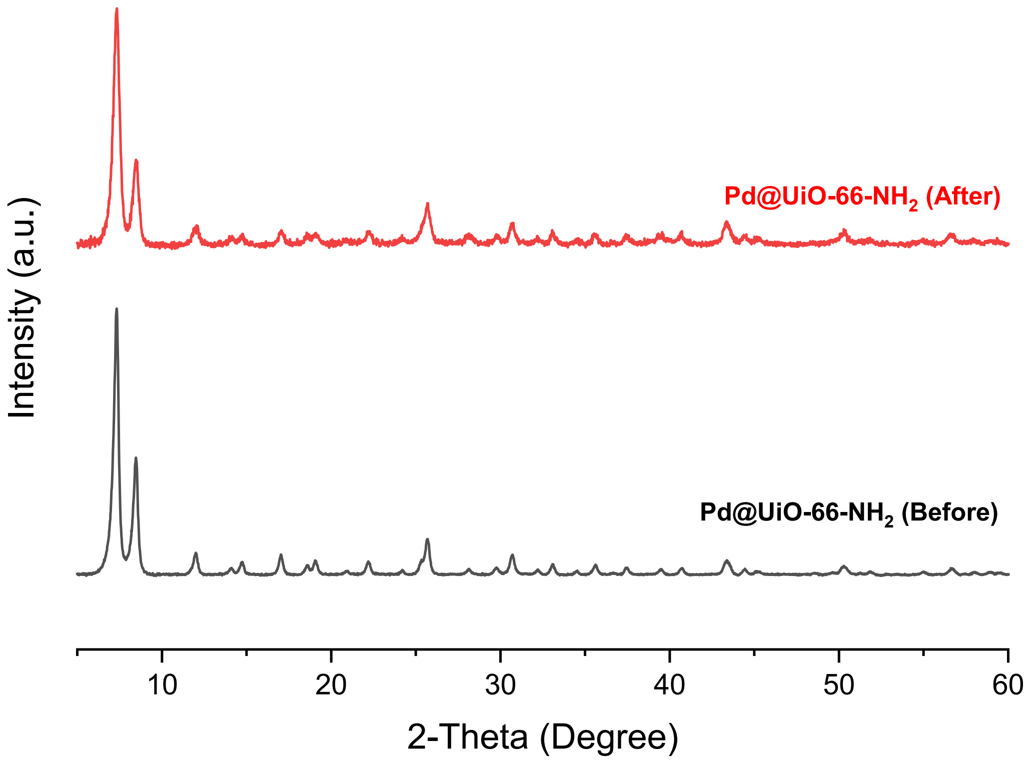

Figure S10. PXRD patterns of Pd(1.7%)@UiO-66-NH_2 before and after the hydrogenation of 1-hexene

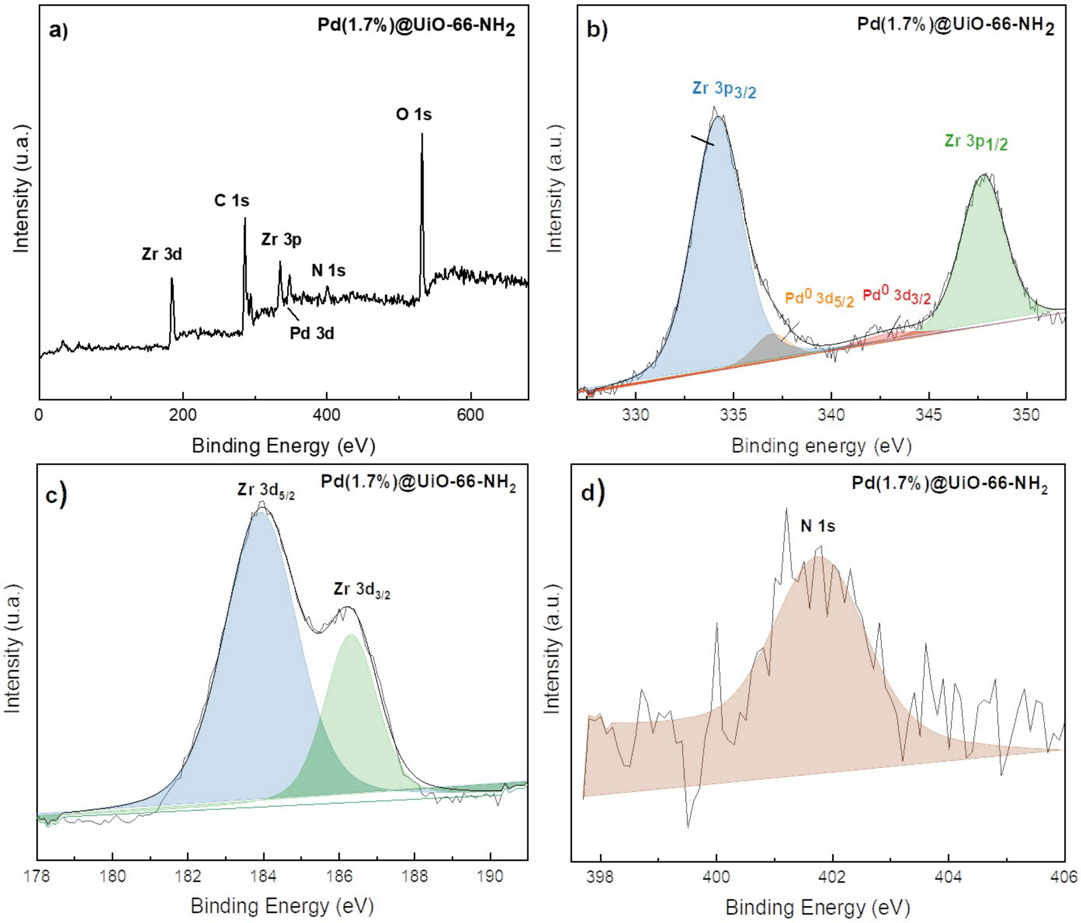

Figure S11. XPS spectra of Pd(1.7%)@UiO-66-NH_2 after the hydrogenation of 1-hexene a) survey, b) Zr 3p and Pd 3d regions, c) Zr 3d region and d) N1s region.

Table S2. Comparison of catalytic hydrogenation activity of MOF-based composites

Composite	Reactant	Metal loading (wt%)	Time (h)	Solvent	H_2 source	TOF (h^{-1})[a]
Pd/C_3N_4 [3]	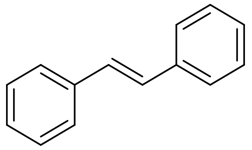	1.5	4	EtOH	NH_4COOH	133
Pd@silica-NH_2[4]	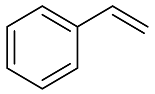	5	0.5	KOH (0.2 M)	H_2 (1 atm)	128
Pd/UiO-66[5]		5	15	EtOH		127
Pd/CuBTC[6]		1	1.5	Toluene-d_8		926
Pd/UiO-66[7]	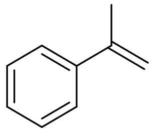	1.6	15	EtOH	1 atm H_2 balloon	456
Pt/UiO-66[8]	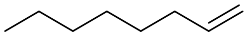	2	24	EtOAc	H_2 (1 atm)	31.4
Pt@UiO-66-NH_2[9]	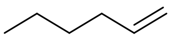	2	1			500
Pt@ZIF-8-"sur"[10]		0.9	12			30
Pt@ZIF-8-"sur"[10]		0.9	12			1337[b]
Pt@UiO-66-NH_2[11]		1.5	1			1007
Pt/UiO-66[12]		1.7	1			770
Pt/MIL-88b (Cr)[12]		1.4	1			1052
Pt/NiBDC (Ni)[12]		1.3	1			989
**Pd@UiO-66-NH_2[c]**		**1.7**	**1**			**3130**

[a]TOF calculated by moles of converted olefin per moles of metal per hour; [b]TOF calculated by moles of converted olefin *per* moles of active sites *per* hour [c] Pd(1.7%)@UiO-66-NH_2 obtained in this work (TOF = mol of product (hexane) *per* mol of metal *per* hour).

(3) Li, J.; Cheng, S.; Du, T.; Shang, N.; Gao, S.; Feng, C.; Wang, C.; Wang, Z. Pd Anchored on C3N4 Nanosheets/reduced Graphene Oxide: An Efficient Catalyst for the Transfer Hydrogenation of Alkenes. *New J. Chem.* **2018**, *42* (11), 9324–9331.

(4) Shabbir, S.; Lee, S.; Lim, M.; Lee, H.; Ko, H.; Lee, Y.; Rhee, H. Pd Nanoparticles on Reverse Phase Silica Gel as Recyclable Catalyst for Suzuki-Miyaura Cross Coupling Reaction and Hydrogenation in Water. *J. Organomet. Chem.* **2017**, *846*, 296–304.

(5) Li, X.; Goh, T. W.; Li, L.; Xiao, C.; Guo, Z.; Zeng, X. C.; Huang, W. Controlling Catalytic Properties of Pd Nanoclusters through Their Chemical Environment at the Atomic Level Using Isoreticular Metal–Organic Frameworks. *ACS Catal.* **2016**, *6* (6), 3461–3468.

(6) Fan, Z.; Staiger, L.; Hemmer, K.; Wang, Z.; Wang, W.; Xie, Q.; Zhang, L.; Urstoeger, A.; Schuster, M.; Lercher, J. A.; Cokoja, M.; Fischer, R. A. Enhanced Catalytic Performance of Palladium Nanoparticles in MOFs by Channel Engineering. *Cell Reports Phys. Sci.* **2022**, *3* (2), 100757–100770.

(7) Tong, L.; Song, X.; Hua, Z.; Zhao, B.; Li, Y. Efficiently Catalytic Transfer Hydrogenation and Fast Separation of Unsaturated Alkene Compounds over Pd/UiO-66 under Green Conditions. *Appl. Catal. A Gen.* **2022**, *643*, 118755.

(8) Zhang, W.; Lu, G.; Cui, C.; Liu, Y.; Li, S.; Yan, W.; Xing, C.; Chi, Y. R.; Yang, Y.; Huo, F. A Family of Metal-Organic Frameworks Exhibiting Size-Selective Catalysis with Encapsulated Noble-Metal Nanoparticles. *Adv. Mater.* **2014**, *26* (24), 4056–4060.

(9) Liu, H.; Chang, L.; Bai, C.; Chen, L.; Luque, R.; Li, Y. Controllable Encapsulation of "Clean" Metal Clusters within MOFs through Kinetic Modulation: Towards Advanced Heterogeneous Nanocatalysts. *Angew. Chemie Int. Ed.* **2016**, *55* (16), 5019–5023.

(10) Yang, Q.; Liu, W.; Wang, B.; Zhang, W.; Zeng, X.; Zhang, C.; Qin, Y.; Sun, X.; Wu, T.; Liu, J.; Huo, F.; Lu, J. Regulating the Spatial Distribution of Metal Nanoparticles within Metal-Organic Frameworks to Enhance Catalytic Efficiency. *Nat. Commun.* **2017**, *8* (1), 14429–14437.

(11) Meng, F.; Zhang, S.; Ma, L.; Zhang, W.; Li, M.; Wu, T.; Li, H.; Zhang, T.; Lu, X.; Huo, F.; Lu, J. Construction of Hierarchically Porous Nanoparticles@Metal-Organic Frameworks Composites by Inherent Defects for the Enhancement of Catalytic Efficiency. *Adv. Mater.* **2018**, *30* (49), 1803263.
(12) Qin, P.; Yan, J.; Zhang, W.; Pan, T.; Zhang, X.; Huang, W.; Zhang, W.; Fu, Y.; Shen, Y.; Huo, F. Prediction Descriptor for Catalytic Activity of Platinum Nanoparticles/Metal–Organic Framework Composites. *ACS Appl. Mater. Interfaces* **2021**, *13* (32), 38325–38332.

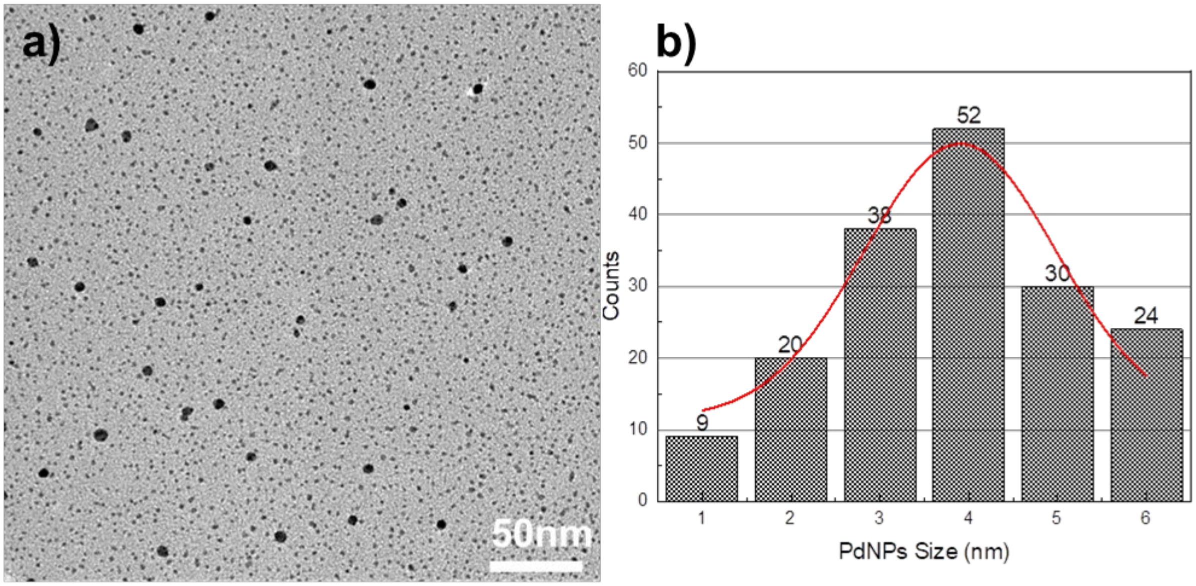

Figure S12. a) TEM image and b) Pd particle size distribution (average size of around 4 nm) of PVP-stabilized Pd nanoparticles.

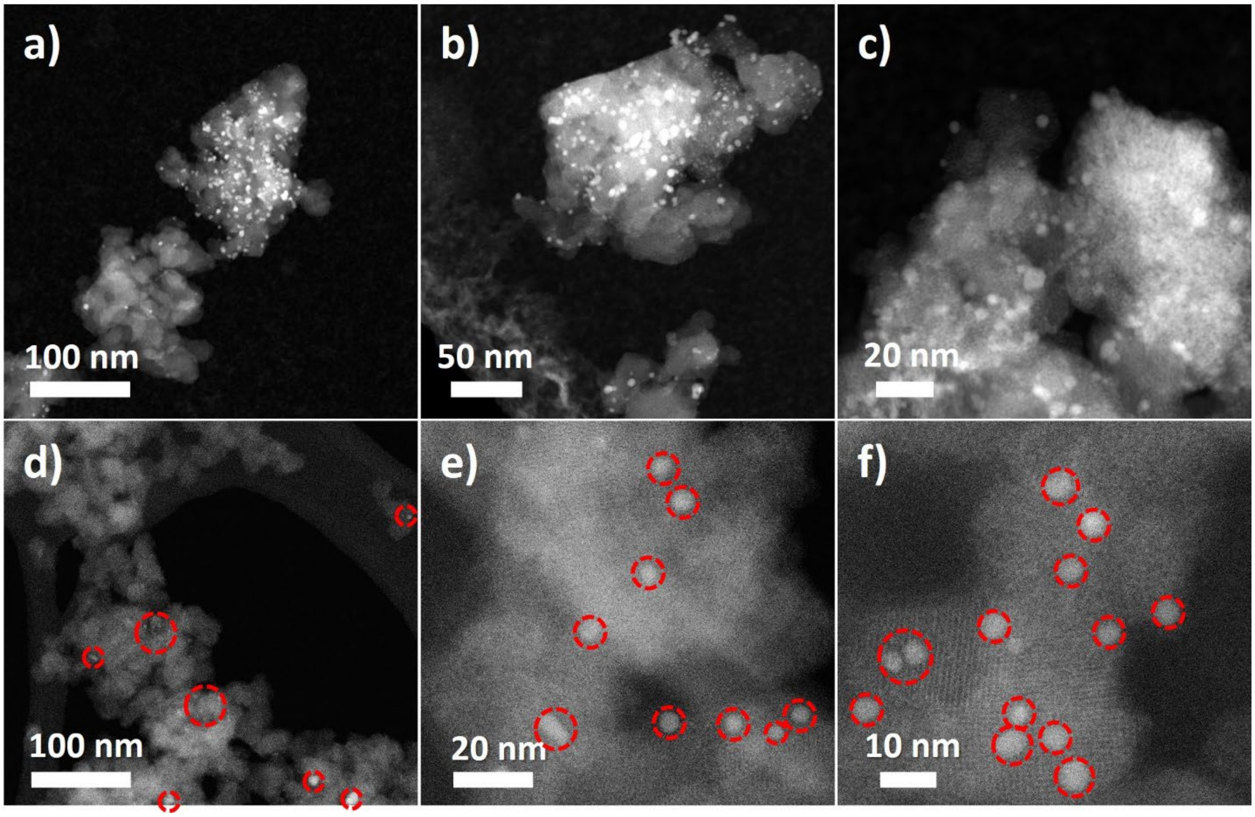

Figure S13. HAADF-STEM images of Pd(1.7%)@UiO-66-NH_2 before oxidative treatment (a, b and c) and after oxidative treatment (d, e and f). Red circles indicate examples of Pd NP locations.

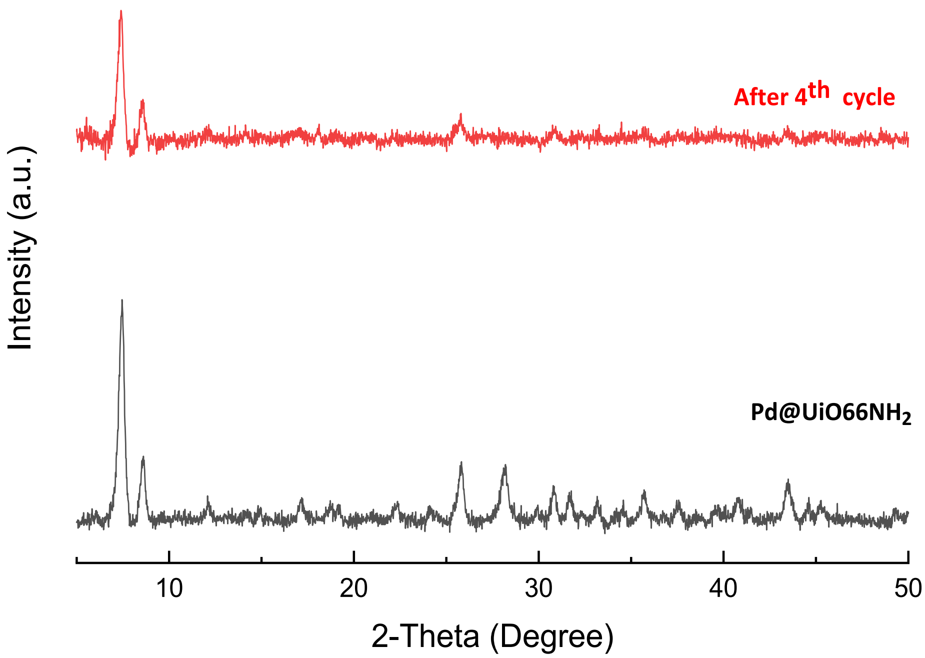

Figure S14. PXRD patterns of Pd(1.7%)@UiO-66-NH_2: fresh and after 4 cycles of hydrogenation of 1-hexene

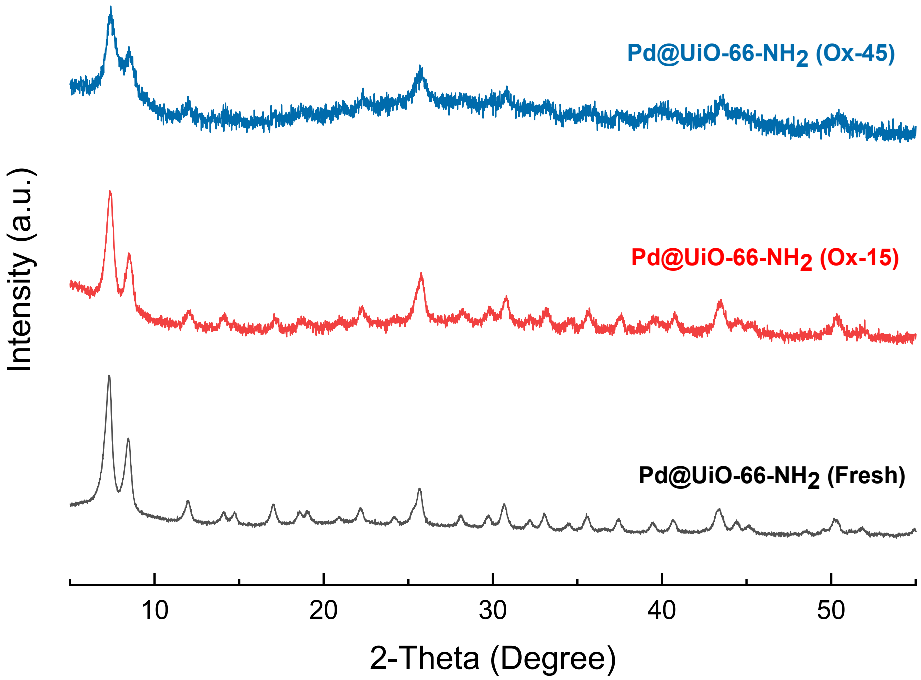

Figure S15. PXRD patterns of Pd(1.7%)@UiO66-NH_2: fresh (black), after oxidative treatment for 15 (Ox-15) (red) and 45 min (Ox-45) (blue)

^{1}H NMR spectrum of tridecane ($CDCl_3$)

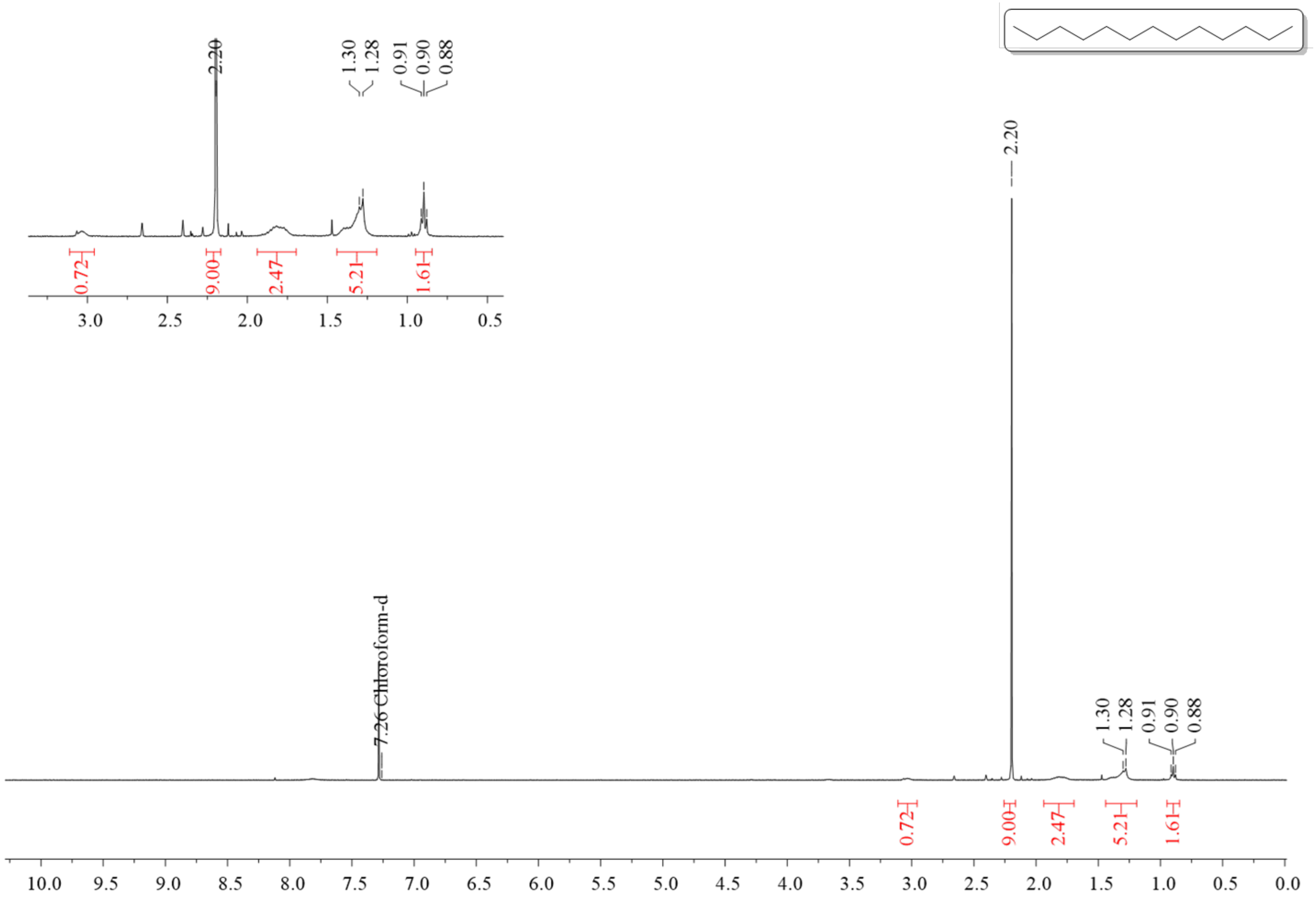

^{13}C NMR spectrum of tridecane ($CDCl_3$)

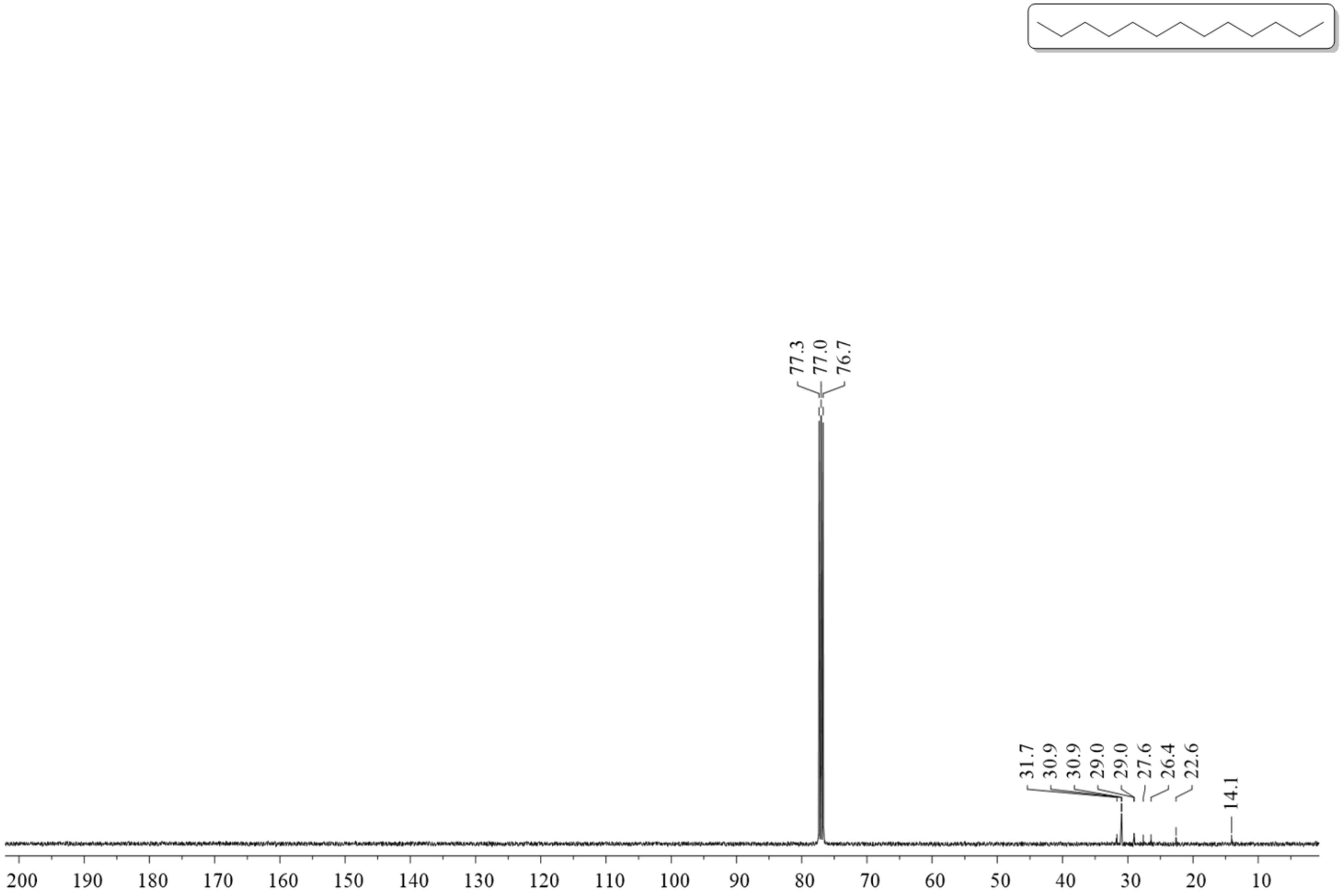

^{1}H NMR spectrum of 1,1,2,2-tetraphenylethane ($CDCl_3$)

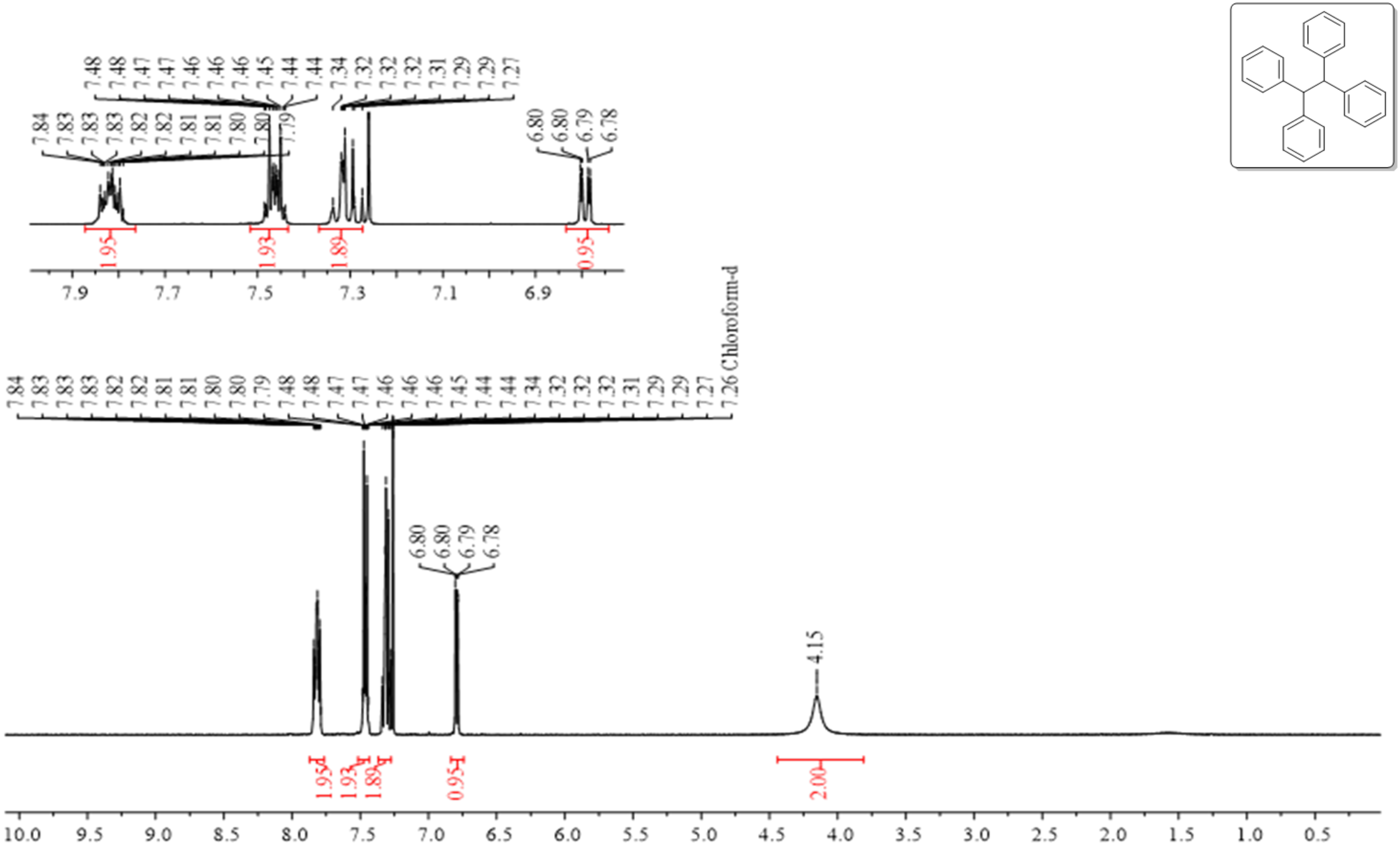

^{13}C NMR spectrum of 1,1,2,2-tetraphenylethane ($CDCl_3$)

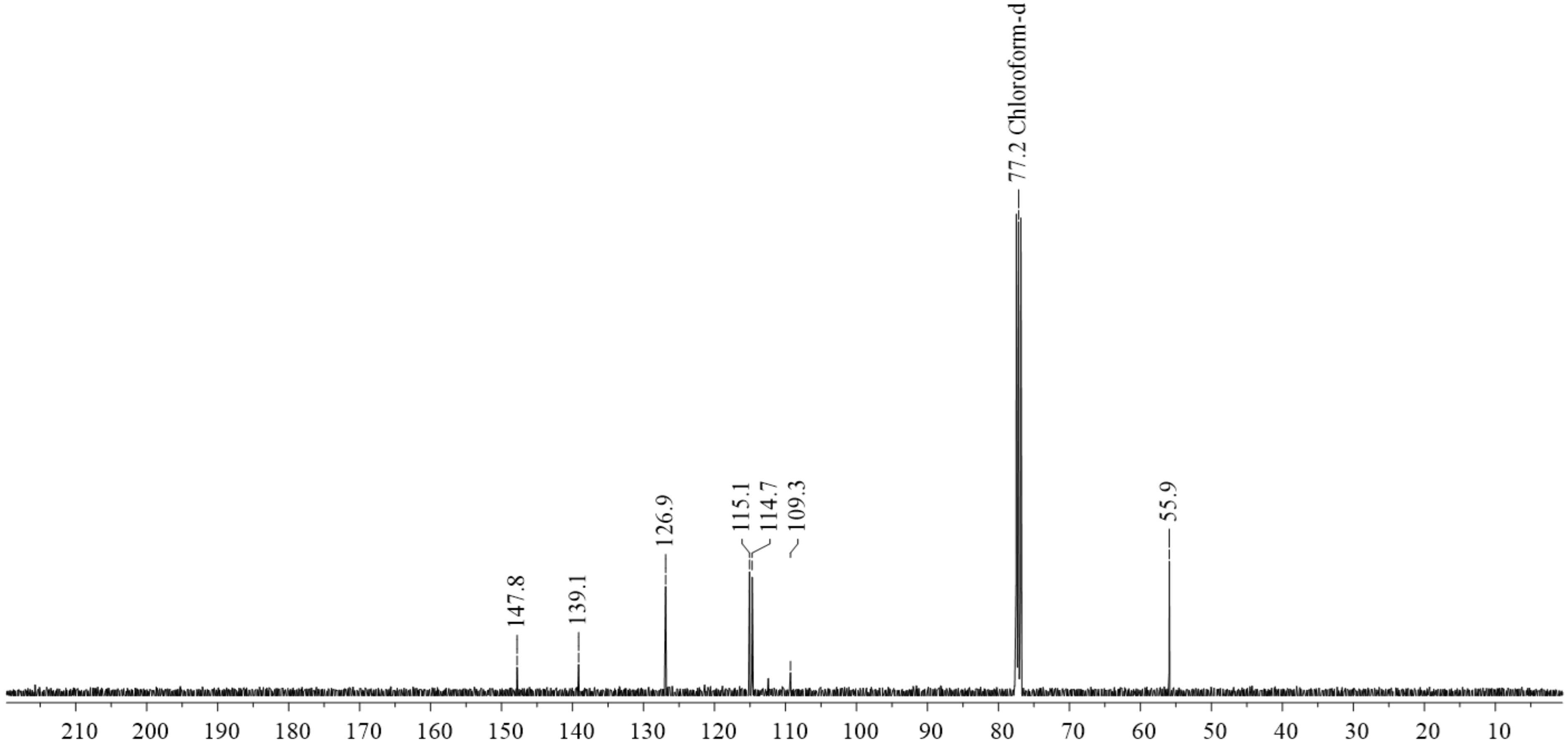

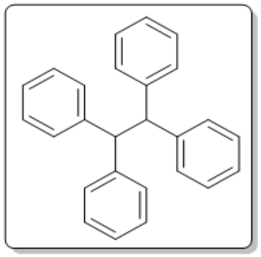

Supplement: Supplementary file 1 — am4c03106_si_001.pdf [file am4c03106_si_001.pdf]
